# Systematic expression alteration analysis of master reprogramming factor OCT4 and its three pseudogenes in human cancer and their prognostic outcomes

**DOI:** 10.1038/s41598-018-33094-7

**Published:** 2018-10-04

**Authors:** Subbroto Kumar Saha, Yeojin Jeong, Sungha Cho, Ssang-Goo Cho

**Affiliations:** Department of Stem Cell and Regenerative Biotechnology, Incurable Disease Animal Model & Stem Cell Institute (IDASI), Konkuk University, Seoul, 05029 Republic of Korea

## Abstract

OCT4 is a master transcription factor that regulates the pluripotency of pluripotent stem cells and cancer stem cells along with other factors, including SOX2, KLF4, and C-MYC. Three different transcripts, *OCT4A*, *OCT4B*, and *OCT4B1*, are known to be generated by alternative splicing and eight *OCT4* pseudogenes have been found in the human genome. Among them, we examined *OCT4* and three pseudogenes (*POU5F1P1*, *POU5F1P3*, and *POU5F1P4*) because of their high expression possibility in cancer. In addition, previous studies indicated that *OCT4* expression is augmented in cervical cancer and associated with poor prognosis, whereas *OCT4* is down-regulated and correlated with good clinical outcomes in breast cancer. Because of these conflicting reports, we systematically evaluated whether expression of OCT4 and its pseudogenes can serve as oncogenic markers in various human cancers using the Oncomine database. Moreover, copy number alterations and mutations in *OCT4* gene and its pseudogenes were analyzed using cBioPortal and the relationship between expression of *OCT4* and pseudogenes and survival probability of cancer patients were explored using Kaplan-Meier plotter, OncoLnc, PROGgeneV2, and PrognoScan databases. Multivariate survival analysis was further conducted to determine the risk of the expression of the occurrence of *OCT4* and its pseudogenes on certain cancer types using data from the Kaplan-Meier plotter. Overall, an association between expression of *OCT4* and pseudogenes and cancer prognosis were established, which may serve as a therapeutic target for various human cancers.

## Introduction

Approximately 14.1 million new cancer cases and 8.2 million deaths occurred worldwide in 2012^1^ and, by 2030, the universal burden is anticipated to increase to 21.7 million new cancer cases and 13 million cancer deaths because of aging and growth of the population^2,3^. Thus, cancer has become a major cause of death for humans. Cancer occurrence has various causes, all of which are related to a specific class of genes called proto*-*oncogenes or oncogenes. A proto-oncogene can be transformed into an oncogene. Activation of a proto-oncogene into an oncogene can occur through a point mutation, gene amplification, or gene translocation^4^. These mutations can alter the DNA copy number and gene function at various locations of a specific genome^5–7^. Pollack *et al*. reported that copy number alterations (CNAs) affect gene expression, which may be a critical component of tumor progression^7,8^. Detecting CNAs may enable researchers to relate a CNA with a disease phenotype^7,9^, providing a basis for clinicians and scientists to identify new biomarkers or signaling pathways in cancer for therapeutics development or early interference to prevent cancer^7^.

A transcription factor (TF), octamer-binding transcription factor 4 (*OCT4*), also known as POU (Pituitary-specific Pit-1, Octamer (ATGCAAAT) transcription factor, and neural Unc-86 transcription factor) domain class 5 Homeobox transcription factor 1 (POU5F1), regulates the pluripotency of pluripotent stem cells^10^. *OCT4* was also reported to be highly expressed in several other types of cancer cells. However, numerous previous reports showed opposite functions of the *OCT4* gene; some studies revealed that under-expression of *OCT4* inhibited cell proliferation or metastasis in different types of cancer cells^11,12^, while another study showed that *OCT4* over-expression suppressed metastasis in breast cancer cells^12^. Thus, the reprogramming factor OCT4 may differentially regulate cancer properties. Three transcript variants, *OCT4A*, *OCT4B*, and *OCT4-B1*, can be generated from *OCT4* by alternative splicing and its eight pseudogenes are generated from different chromosomes. Each variant of *OCT4* produces distinct mRNA sequences and proteins and function differently in cancer cells^11–16^. Among them, OCT4A is confined to the nucleus of embryonic stem cells (ESCs), embryonic carcinoma, cancer stem and germinal cells, and germ cell tumors where it acts as the key TF to maintain the self-renewal and pluripotency of the cells^12,17–21^. In contrast, OCT4B is predominantly expressed in the cytoplasm of cancer cells and is incapable of sustaining the pluripotency of stem cells. Recent studies revealed the presence of an internal ribosome entry site (IRES) for OCT4B, which can generate three isoforms (OCT4B-164, OCT4B-190, and OCT4B-265) by alternative translation initiation^12,17–22^. Another variant, OCT4B1, is localized in both the cytoplasm and nucleus of undifferentiated and pluripotent cells^12,14,23,24^. However, OCT4B1 is not considered as a stemness marker. Eight pseudogenes of *OCT4* have been identified: *POU5F1P1*, *POU5F1P2*, *POU5F1P3*, *POU5F1P4*, *POU5F1P5*, *POU5F1P6*, *POU5F1P7*, and *POU5F1P8*^16,19^.

Currently, approximately 20,000 pseudogenes have been identified in the human genome^8,25^. In the past several years, pseudogenes have been referred to as “genomic fossils” and treated as “junk DNA”. However, several pseudogenes were shown to play important roles in gene regulation of their parental genes, and numerous pseudogenes are transcribed into RNA^26^. Additionally, some pseudogenes cause gene silencing and thus control the expression of their parent genes. In contrast, several transcribed pseudogenes are translated to produce antigenic peptides or truncated proteins. These results indicate that pseudogenes are not junk DNA and have vital functions within normal and abnormal cells^12,27–29^. It remains unclear whether pseudogene translation affects cell. However, the transcription of pseudogenes has different effects in various cancer types. Thus, *OCT4* pseudogenes may also be transcribed and influence various cancer phenotypes.

In this study, we systematically evaluated whether *OCT4* and its pseudogenes are associated with various human cancers and their prognostic outcomes using various oncogenic portals. We used Oncomine and TCGA databases to evaluate gene expression, mutations, and copy number alterations; PrognoScan, OncoLnc, Kaplan-Meier plotter, and PROGgeneV2 databases were used to predict prognostic outcomes. We also retrieved co-expressed genes from TCGA database and analyzed this information using DAVID functional annotation tools to predict probable signaling pathways involved in various cancers. Taken together, our systematic analysis for *OCT4* and expression of its pseudogenes may reveal the association between cancer progression and clinical prognosis, which can be used to develop therapeutic approaches for various human cancers.

## Results

### Transcript expression pattern of *OCT4* and its pseudogenes

**Figure 1:**
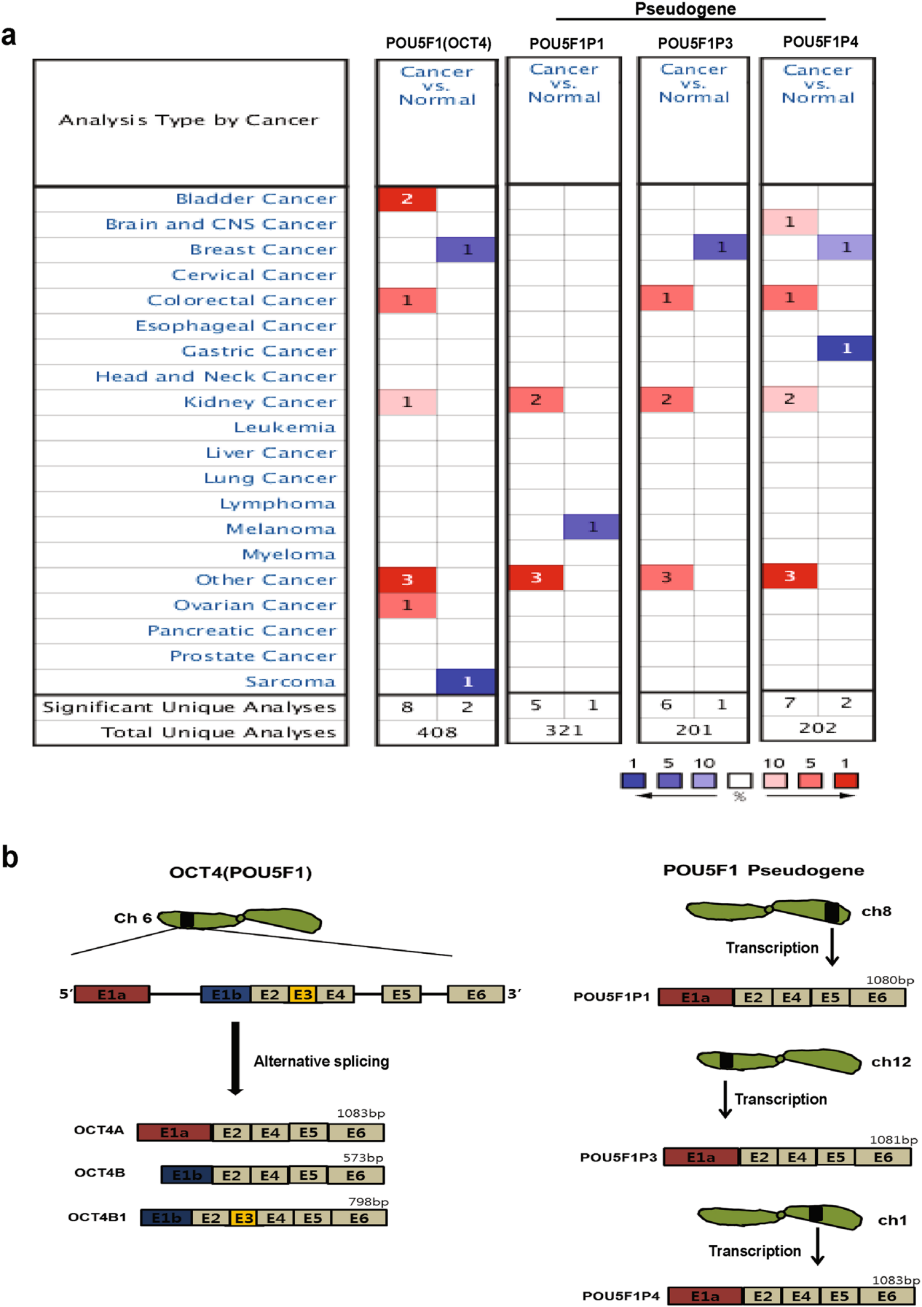
mRNA expression pattern of *OCT4* and its pseudogenes in various cancer types: (**a**) The comparison indicated the number of datasets with *OCT4* and its pseudogene mRNA over-expression (right column, red) and under-expression (left column, blue) in cancer versus normal tissues. The threshold was designed with the following parameters: *p*-value of 1E-4, fold-change of 2, and gene ranking of 10%. (**b**) Schematic view of *OCT4* and its pseudogene transcription. *POU5F1* and its three pseudogenes (*POU5F1P1*, *POU5F1P3*, and *POU5F1P4*) showed high homology in their mRNA sequences, but each gene was in a different chromosome.

OCT4 (POU5F1) plays a crucial role in the maintenance of pluripotency of stem cells and in generating induced pluripotent stem cells (iPSCs). *OCT4* has three known transcript variants and eight pseudogenes^12,15,16^. Among the eight pseudogenes, we selected three pseudogenes (*POU5F1P1*, *POU5F1P3*, and *POU5F1P4*) along with *OCT4* for bioinformatics analysis because of their expression availability in various cancers (Fig. 1a). There are also three known variants of *OCT4* (*OCT4A*, *OCT4B*, and *OCT4B1*), which originate from alternative splicing or different promoters. The *OCT4A*, *OCT4B*, and *OCT4B1* transcripts are distinguished by their first exon sequence. Additionally, as compared to *OCT4B*, the *OCT4B1* transcript contain only more intron 2 than the *OCT4B* transcript^12,15,24^. *OCT4* is in chromosome 6^30^, while three pseudogenes are respectively found at different chromosome sites and transcribed mRNA, which is similar to *OCT4A* (Fig. 1b). However, *OCT4* and its three pseudogenes individually show different expression patterns and differentially affect various cancer types. To confirm the role of *OCT4* and its pseudogenes, we first systemically analyzed mRNA expression between normal tissues and various tumor tissues using the Oncomine database (Fig. 1a). The threshold was designated according to the following values: *p*-value 1E-4, fold-change 2, and top gene ranks 10%^7^. Compared to that in normal tissue, *OCT4* expression was higher in several cancer types except breast cancer and sarcoma. Not only *OCT4* but also its three pseudogenes appeared to be differentially expressed in various cancers (Fig. 1a). Particularly, in breast cancer, expression of *OCT4*, *POU5F1P3*, and *POU5F1P4* was down-regulated compared to in normal tissue. However, all four genes (*OCT4* and three pseudogenes) were expressed higher in the kidney and other cancer types than in their normal tissue. These data indicate that OCT4 and its three pseudogenes function as either oncogene or tumor suppressor in several cancer types. The comprehensive systematic analysis of *OCT4* and its three pseudogenes (*POU5F1P1*, *POU5F1P3* and *POU5F1P4*) are described below.

### OCT4 (POU5F1)

**Figure 2:**
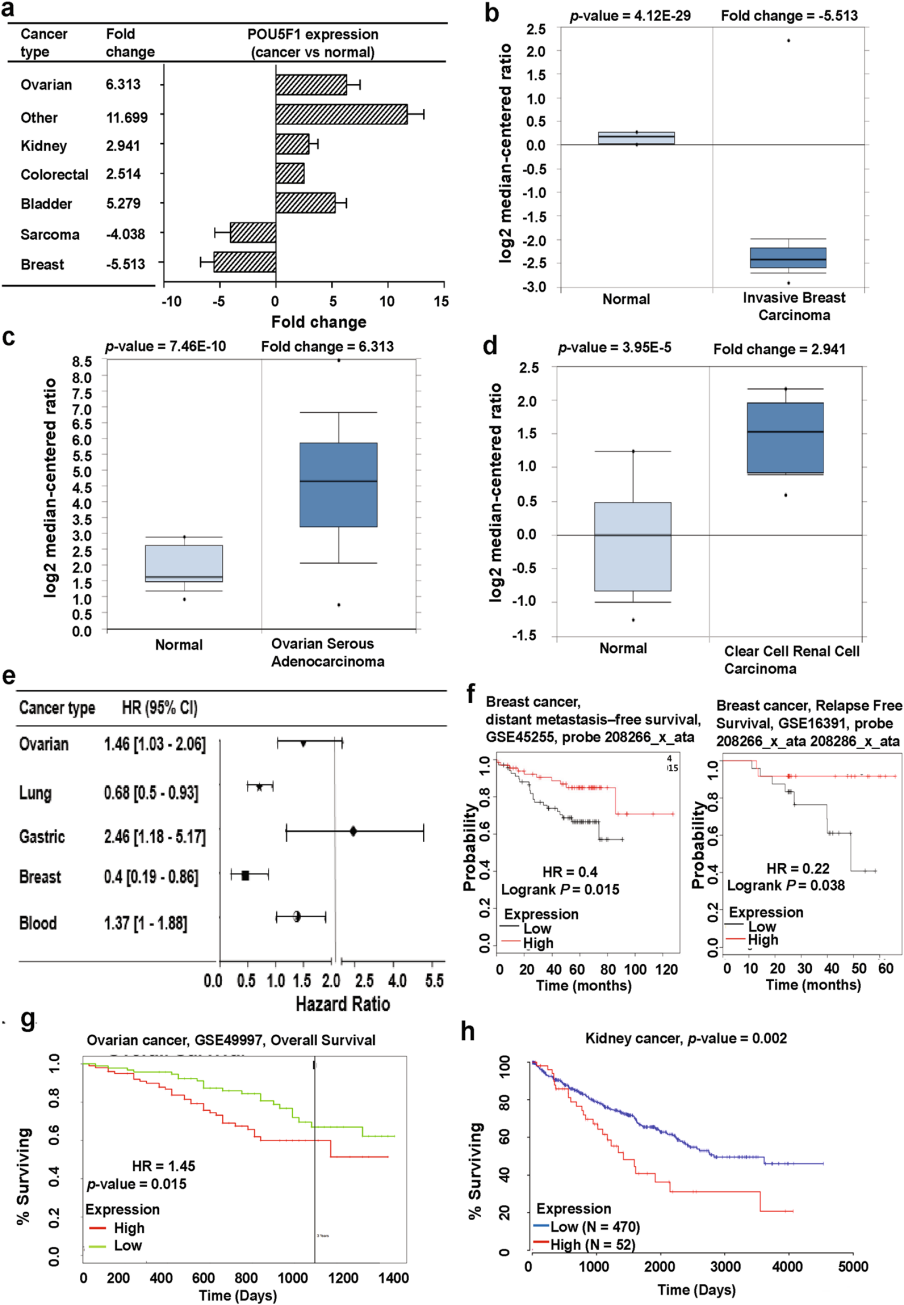
*OCT4* (*POU5F1*) expression pattern and patient survival analysis in different cancer types, compared to *OCT4* expression in normal tissue and each cancer tissue. (**a**) The fold-change of *OCT4* in various types of cancers was identified by our analyses as shown in Supplementary Table S1 and expressed as a forest plot. (**b–d**) The box plot comparing specific *OCT4* expression in normal (left plot) and cancer tissue (right plot) was derived from the Oncomine database. The analysis was shown in breast carcinoma relative to in normal breast (**b**), in ovarian adenocarcinoma relative to in normal ovarian tissue (**c**), in renal carcinoma relative to normal renal (**d**). (**e**) Significant hazard ratios in various types of cancers were identified from our analyses shown in Supplementary Table S2 and expressed as a forest plot. (**f–h**) The survival curve comparing patients with high (red) and low (black, blue, and green) expression in breast (**f**), ovarian (**g**), kidney (**h**) cancer was plotted from the Kaplan Meier-plotter, PROGgeneV2, and OncoLnc database. Survival curve analysis was conducted using a threshold Cox *p*-value < 0.05.

**Figure 3:**
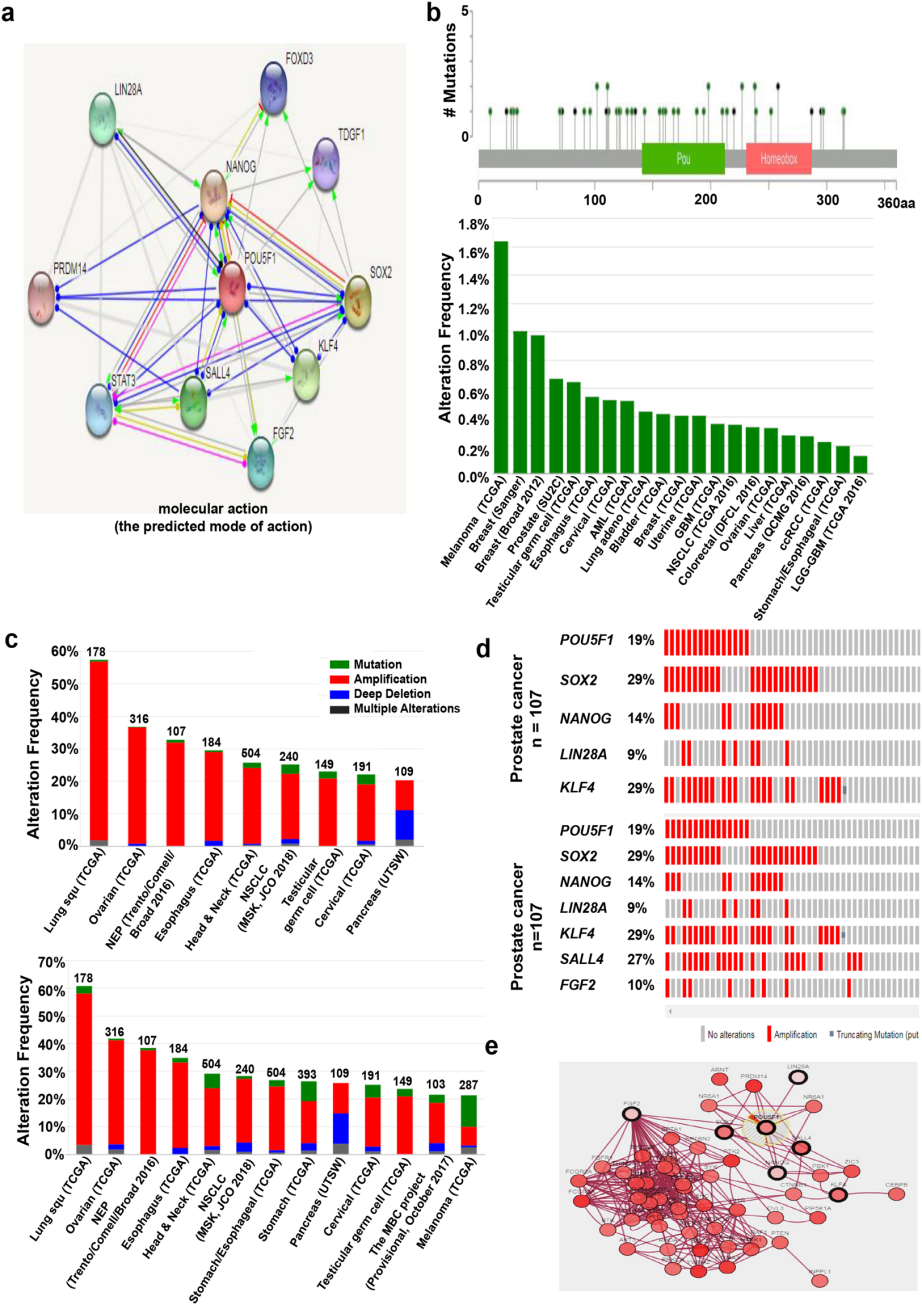
Mutation and alteration frequency patterns of *OCT4* (*POU5F1*) and its associated genes in various cancers: (**a**) Functional protein partner of OCT4 was predicted by STRING. Line indicates the predicted mode of molecular action. (**b**) Mutation diagram of POU5F1 in different cancer types across protein domains. POU5F1 mutation frequencies are the highest in melanoma and POU5F1 mutation mere more frequent in N-domain than in the C-domain. (**c**) The alteration frequency of a five-gene signature (*POU5F1*, *SOX2*, *NANOG*, *LIN2BA*, and *KLF4*) was determined using cBioPortal and is shown on the top. The alteration frequency of a seven-gene signature (*POU5F1*, *SOX2*, *NANOG*, *LIN2BA*, *KLF4*, *SALL4*, and *FGF2*) was determined using cBioPortal and is shown on the bottom. Only cancer types containing >100 samples and an alteration frequency of >20% are shown. The alteration frequency included deletions (blue), amplification (red), multiple alterations (grey), or mutation (green). The total number of samples for each cancer type is indicated by the numbers at the top of each column. Prostate cancer types frequently amplify *POU5F1*. We used the Oncoprint feature of cBioPortal to determine the copy number alteration frequency of each gene in *POU5F1* within selected cancer subtypes. (**d**) The percentages of alterations in five genes and seven genes in the prostate cancer. Grey bars along a vertical line represent the same sample evaluated for amplification (red), deep deletion (blue), missense mutation (green), truncating mutation (black), or in-frame mutation (brown). (**e**) The interactions between *POU5F1* and its associated gene alterations were searched in cBio Cancer Genomics Portal. Network view of the *POU5F1* neighborhood in prostate cancer. Darker red indicates increased frequency of alteration (defined by mutation, copy number amplification, or homozygous deletion) in prostate cancer.

*OCT4* recognized as *POU5F1* is a homeodomain transcription factor of the POU family. It is primarily involved in the self-renewal of undifferentiated stem cells such as iPSCs and ESCs along with cancer stem cells. Recently, several studies showed that *OCT4* regulates cancer proliferation and metastasis as well as maintains pluripotency in undifferentiated cells. *OCT4* was reported to play an important role in tumorigenesis and has been suggested as a prognostic prediction marker for testicular germ cell tumor (TGCT), and for pancreatic, lung, and liver cancers^31^. Another study revealed that *OCT4* induced tumorigenesis and prevented the apoptosis of cervical cancer cells^11^. In contrast, over-expression of *OCT4* was found to suppress the metastatic potential of breast cancer^12^. Given these contrasting roles of *OCT4* in cancer, we first applied the Oncomine database to identify the expression pattern of *OCT4* in several cancers with significant *p*-values. From the Oncomine data, we found that *OCT4* was up-regulated in bladder, colorectal, kidney, ovarian, and other cancers, but decreased in breast cancer and sarcoma compared to in their normal tissue (Fig. 2a–d; Supplementary Table S1). These data are consistent with those of previously published studies of *OCT4* expression^11,12^. In Fig. 2e, we summarized the prognostic value of *OCT4* expression in various cancers using patient prognosis data from numerous databases with significant Cox *p*-values (*p* < 0.05). Moreover, the Kaplan Meier-plot and PrognoScan showed that low expression of *OCT4* is associated with poor prognosis in breast cancer (Fig. 2f; Supplementary Table S2). In contrast, using the PROGgeneV2 and OncoLnc database, the relationship between over-expression of *OCT4* and low patient survival rates was confirmed in ovarian and kidney cancers (Fig. 2g,h; Supplementary Table S2). Next, we selected the functional protein partners of OCT4 based on active interaction sources including text mining, experiments, and curated databases. The 10 predicted interacting proteins of OCT4 were extracted using protein-protein interaction databases with the STRING v10.5 program (Fig. 3a). The most confident predicted interacting proteins were SOX2 (SRY-box 2), NANOG (Nanog homeobox), LIN2BA (Lin-28 homolog A), KLF4 (Kruppel-like factor 4), SALL4 (Sal-like 4), FGF2 (Fibroblast growth factor 2), PRDM14 (PR domain containing 14), FOXD3 (Forkhead box D3), STAT3 (Signal transducer and activator of transcription 3), and TDGF1 (Teratocarcinoma-derived growth factor 1) (Fig. 3a).

To analyze OCT4 mutations and copy number alterations (CNAs) in various cancer types, we used the cBioPortal web and found that most mutations occurred in the N-terminus and POU domain of the OCT4 protein (Fig. 3b, upper panel). We then investigated the alteration frequency of OCT4 mutations in various cancer types using cBioPortal web. The results showed that OCT4 mutations were increased in several cancer types, particularly in melanoma and breast cancer with alteration frequencies 1.6 and 1, respectively (Fig. 3b, lower panel). There are four main factors involved in cancer development: genetic, epigenetic, transcriptomic, and proteomic alterations^32^. These alterations arise in specific regions of the genomes, revealing their potential oncogenic or suppressive roles^33^. Thus, we focused on significant CNAs in various cancer types using cBioPortal. We selected seven genes signature according to their confidence scores among the 10 predicted functional protein partners of OCT4 such as SOX2, NANOG, LIN28A, KLF4, SALL4, and FGF2 from the STRING database. First, we analyzed the five-gene signatures *OCT4*, *SOX2*, *NANOG*, *LIN28A*, and *KLF4* using cBioPortal. The data was examined in 9 different cancer studies representing 2,882 samples showing a >20% alteration frequency with at least 100 samples in the dataset. The percentage of alterations were 57.3–20.18% in decreasing order (highest to lowest) in the lung, ovarian, prostate, esophagus, head and neck, pan-lung, germ, cervical, and pancreatic cancer (Fig. 3c, upper panel; Supplementary Table S3). In the seven-gene signature analysis, 13 different cancer studies of 3,930 samples met the above-mentioned criteria (Fig. 3c, lower panel; Supplementary Table S4). The percentage of alterations was 60.67–21.25% in various cancer types, while the highest rate of alternation was observed in lung cancer; we focused on prostate cancer because of the predominant pattern of each specific gene amplification. In detail, the alteration percentage of the five-gene signature in prostate cancer varied from 9 to 29%. (*OCT4*, 19%; *SOX2*, 29%; *NANOG*, 14%; *LIN28A*, 9%; *KLF4*, 29%); the *SOX2* and *KLF4* genes were amplified predominantly in prostate cancer (Fig. 3d, upper panel). The amplification percentage of each gene in other cancer cells is summarized in Supplementary Tables S5,6. Additionally, the amplification percentages of *SALL4* (27%) and *FGF2* (10%) were determined when the seven-gene signature was analyzed for prostate cancer type (Fig. 3d, lower panel), indicating that co-expression genes of *OCT4* partially regulates cancer development. The cBioPortal can also be employed for collaborative analysis and visualization of altered networks. The networks contain interactions and pathways from the Human Protein Reference Database^34^, NCI Pathway Interaction Database^35^, Reactome^36^, and MSKCC Cancer Cell Map^37^. Figure 3e shows the network view of the OCT4 neighborhood in neuroendocrine prostate (NEP) cancer. These results improve the understanding of the underlying molecular mechanisms of OCT4 in various cancers.

Co-expression profile of *OCT4* was retrieved from the Oncomine database, which was inferred to define the critical signaling pathways involved (Supplementary Fig. S1). The co-expression profile of *OCT4* was identified across 6 normal and 27 seminoma tissues (Supplementary Fig. S1a), as well as across 10 normal and 43 ovarian cancers samples (Supplementary Fig. S1b). *OCT4* was found to be highly co-expressed with its three pseudogenes (*POU5F1P1*, *POU5F1P3*, and *POU5F1P4*) in seminoma (Supplementary Fig. S1a). These data suggest that the pseudogene of *OCT4* affects the expression of *OCT4*. In reality, *POU5F1P4*, a known *OCT4* pseudogene, regulates *OCT4* expression in hepatocellular carcinoma^38^. Additionally, in ovarian cancer, *OCT4* was co-expressed with *DNAJBI3*, *C2orf88*, and several genes with lower correlation values compared to the three pseudogenes (*POU5F1P1*, *POU5F1P3*, and *POU5F1P4*) at seminoma (Supplementary Fig. S1b).

Next, we retrieved the co-expressed genes of *OCT4* from TCGA database using cBioPortal. GO (gene ontology) analyses of co-expressed genes were performed using DAVID functional annotation tools to reveal the probable potential underlying the signaling pathways related to *OCT4* (Supplementary Fig. S1); the results showed that *OCT4* mostly regulates biosynthesis and metabolism-related pathways in testicular cancer. *OCT4* is predominantly associated with the long-chain fatty acid breakdown (peroxisome) pathway compared to other pathways in testicular cancer (Supplementary Fig. S1a, right panel). By contrast, in ovarian cancer cells, *OCT4* mainly regulates endocytosis and the p53 signaling pathway (Supplementary Fig. S1b, right panel). These systematic insights may lead to therapeutic approaches targeting OCT4 or its underlying cell signals in cancer treatment. However, the detailed principal mechanism through which OCT4 controls cancer progression requires further examination.

### POU5F1P1

**Figure 4:**
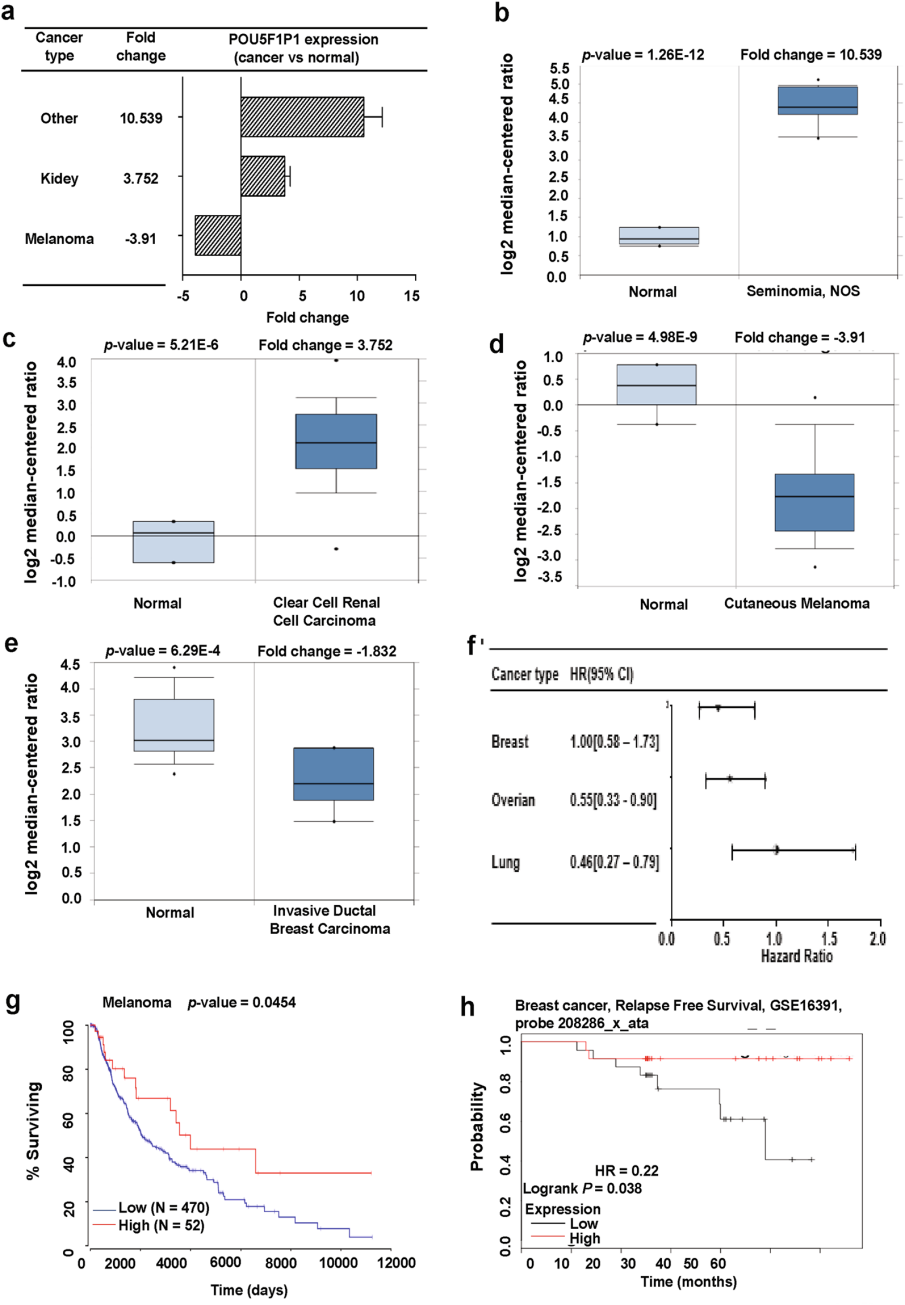
*POU5F1P1* expression and mutation pattern compared to *POU5F1P1* expression in normal tissue and each cancer tissue: (**a**) The fold-change of *POU5F1P1* in various types of cancers was identified from our analyses shown in Supplementary Table S7 and expressed as the forest plot. (**b–e**) The box plot comparing specific *POU5F1P1* expression in normal (left plot) and cancer tissue (right plot) was derived from the Oncomine database. The analysis was shown in seminoma relative to normal testicle (**b**), in renal carcinoma relative to normal renal (**c**), in melanoma relative to normal skin (**d**), and in breast relative to normal breast (**e**). (**f**) Significant hazard ratios in various types of cancers was identified from our analyses shown in Table S8 and expressed as a forest plot. (**g-h**) Survival curve comparing patients with high (red) and low (black, blue) expression in melanoma (**g**), breast (**h**) was plotted from OncoLnc and Kaplan Meier-plotter database. Survival curve was analyzed using a threshold Cox *p*-value < 0.05.

**Figure 5:**
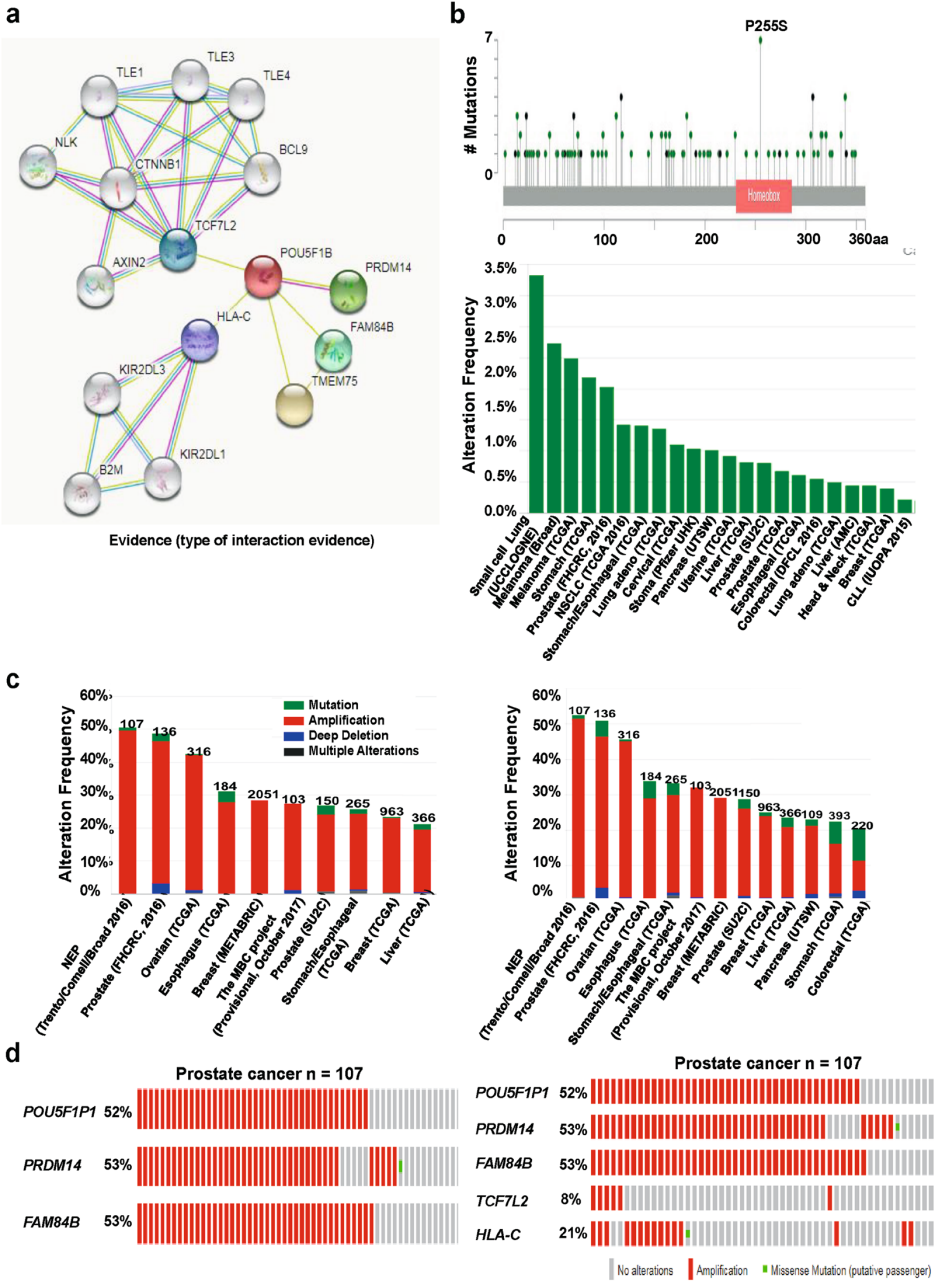
Mutation and alteration frequency patterns of *POU5F1P1* and its associated genes in various cancers. (**a**) Functional protein partner of POU5F1P1 was predicted by STRING web. Line indicates the type of interaction evidence. (**b**) Mutation diagram of POU5F1P1 in different cancer types across protein domains was expressed. POU5F1P1 mutation frequencies are the highest in lung and one hot spots (P255S) representing the common founder mutations in POU5F1P1 homeobox site. The alteration frequency of a three-gene signature (*POU5F1P1*, *PRDM14*, and *FAM84B*) was determined using cBioPortal and is shown on the top. (**c**) The alteration frequency of a five-gene signature (*POU5F1P1*, *PRDM14*, *FAM84B*, *TCFL2*, and *HLA-C*) was determined using cBioPortal and is shown on the bottom. Only cancer types containing >100 samples and an alteration frequency of >20% are shown. The alteration frequency included deletions (blue), amplification (red), multiple alterations (grey), or mutation (green). The total number of samples for each cancer type is indicated by the numbers at the top of each column. Prostate cancer types frequently amplify *POU5F1P1*. We used the Oncoprint feature of cBioPortal to determine the copy number alteration frequency of each gene in *POU5F1* within selected cancer subtypes. (**d**) The percentages of alterations in three genes and five genes in the prostate cancer. Grey bars along a vertical line represent the same sample evaluated for amplification (red), deep deletion (blue), missense mutation (green), truncating mutation (black), or in-frame mutation (brown).

*OCT4-pg1* (octamer-binding transcription factor 4 pseudogene 1), also known as *POU5F1P1*, is one of the eight *OCT4* pseudogenes^13^. Although *POU5F1P1* is generated from different chromosome, not like OCT4, its transcription results in a similar mRNA sequence as *OCT4* (Fig. 1b). The *POU5F1P1* transcript can also produce a 95% homologous protein with OCT4A protein containing N-terminal, C-terminal, and POU domains^12,13,16^. POU5F1P1 is reported to be localized in the nucleus^39^. A presumed POU5F1P1 protein functions as a transcriptional activator and modulates the expression as for OCT4 isoform 1 (OCT4A). However, POU5F1P1 was not a strong activator like OCT4A, perhaps because of the substitution of amino acids from POU5F1P1 protein (but more strongly than OCT4B). It has been reported that *POU5F1P1* strongly increases the risk of colon, prostate cancer, and other cancers^13^. We confirmed the *POU5F1P1* expression pattern in various cancer types using the Oncomine database (Fig. 4a–e; Supplementary Table S7). *POU5F1P1* was over-expressed in seminoma, kidney, and other cancers compared to in their normal tissues (Fig. 4a–c; Supplementary Table S7). Figure 4f also displays the statistically significant prognostic data (p < 0.05) related to the *POU5F1P1* expression pattern in several cancers using various portals for obtaining patient-survival information. Specifically, *POU5F1P1* is down-regulated in melanoma and breast cancer compared to in their normal counterparts, which is significantly associated with a poor prognosis of patient survival (Fig. 4g,h). The association of *POU5F1P1* expression and survival in several cancer patients is summarized in Supplementary Table S8 for the PrognoScan database results. We then confirmed the functional partners of POU5F1P1 through using the STRING database. We selected the potential protein partners of POU5F1P1 based on experiments, text mining, and curated databases using the STRING v10.5 program (Fig. 5a). Additionally, POU5F1P1 mutation predominantly occurred in small cell lung cancer and is in a hotspot in the POU5F1P1 homeobox domain (P255S) (Fig. 5b). The *POU5F1P1* mutation frequencies were graphed for at least 100 samples in the dataset and more than seven cancer types showed a more than 1% mutation alteration frequency (Fig. 5b). We then chose four proteins based on their confidence scores among many predicted proteins to analyze the amplification patterns. The selected proteins were PRDM14 (PR domain containing 14), FAM84B (Family with sequence similarity 84, member B), TCF7L2 (Transcription factor 7-like 2), and HLA-C (Major histocompatibility complex, class I, C) (Fig. 5c,d; Supplementary Tables S9–S12). The results considering the three-gene signature (*POU5F1P1*, *PRDM14*, and *FAM84B*) analyzed in 10 different cancer studies consisting 4,641 samples were limited to more than a 20% alteration frequency and minimum of 100 samples in the dataset. The results from the five-gene signature (adding two genes: *TCF7L2* and *HLA-C*) showed 13 different cancer studies (Fig. 5c). Both gene signature results indicated the highest amplification frequency in NEP cancer with values of 52.34% and 54.21%, respectively. In this cancer type, the specific amplification pattern of individual gene is summarized in Fig. 5d, which was *POU5F1P1*, 52%; *PRDM14*, 53%; *FAM84B*, 53%; *TCF7L2*, 8%; *HLA-C*, 21%. The alteration frequency and each gene alteration percentage in various cancers are shown in Supplementary Tables S9–S12. Next, we focused on genes co-expressed with *POU5F1P1* using the Oncomine database that may be critical for defining pathways. The co-expression profile of *POU5F1P1* was identified across 10 normal and 10 kidney cancer tissues, 7 normal and 45 melanoma samples, and 13 normal and 41 head-neck cancer tissues (Supplementary Fig. S2). Specifically, POU5F1P1 was correlated with RAB27A, a Ras-related protein in kidney cancer, which is commonly expressed in cancer (Supplementary Fig. S2a). Recent experiments showed that Rab27a leads to MVE (multivesicular endosomes) docking at the plasma membrane^40^. In addition, several genes that are co-expressed with *POU5F1P1* in melanoma and head-neck cancer are presented in Supplementary Fig. S2b,c (left panel). Predictive underlying signaling pathways associated with *POU5F1P1* were analyzed by DAVID functional annotation tools (Supplementary Fig. S2). *POU5F1P1* is predominantly involved in several cellular and molecular signaling pathways; the lysosome pathway is most likely involved in melanoma (Supplementary Fig. S2b, right panel). Moreover, *POU5F1P1* affects the cell cycle in kidney cancer and the oxytocin signaling pathway in head-neck cancer (Supplementary Fig. S2a,c; right panel). However, the detailed underlying mechanism of cancer progression regarding *POU5F1P1* expression modulation requires further investigation.

### POU5F1P3

**Figure 6:**
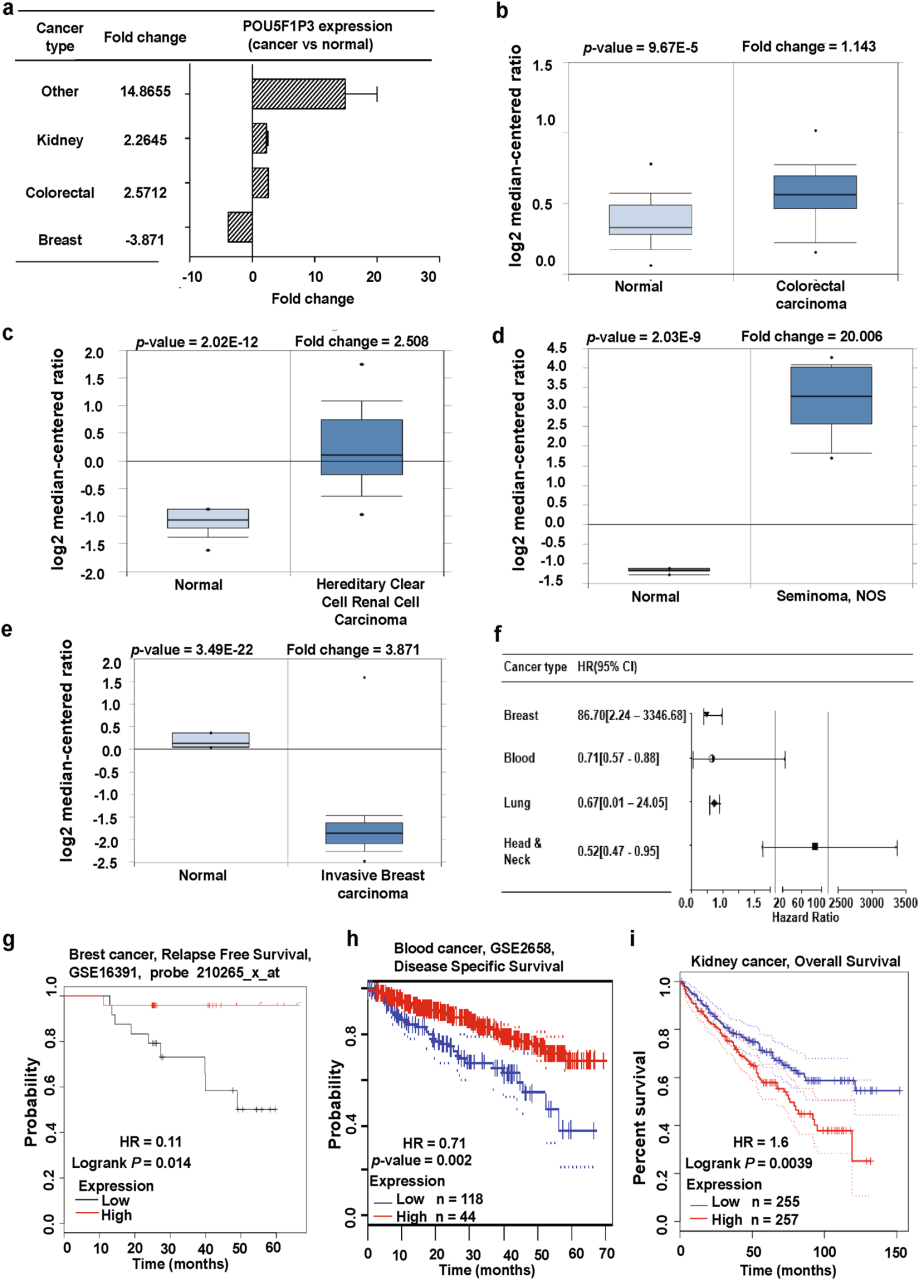
*POU5F1P3* expression pattern and patient survival analysis in different cancer types compared to *POU5F1P3* expression in normal tissue and each cancer tissue. (**a**) The fold-change of *POU5F1P3* in various types of cancers was identified from our analyses shown in Supplementary Table S13 and expressed as the forest plot. (**b–e**) The box plot comparing specific *POU5F1P3* expression in normal (left plot) and cancer tissue (right plot) was derived from the Oncomine database. The analysis was shown in colorectal carcinoma relative to normal colorectal (**b**), in renal carcinoma relative to normal renal (**c**), in seminoma relative to normal testicle (**d**), in breast carcinoma relative to normal breast (**e**). (**f**) Significant hazard ratios in various types of cancers was identified from our analyses shown in Table S14 and expressed as a forest plot. (**g–i**) The survival curve comparing patients with high (red) and low (black, blue) expression in breast (**g**), blood (**h**), and kidney (i) cancers was plotted from the Kaplan Meier-plotter, PrognoScan, and GAPIA database. The survival curve was analyzed using a threshold Cox *p*-value < 0.05.

*OCT4-pg3* (octamer-binding transcription factor 4 pseudogene 3), also known as *POU5F1P3*, is in chromosome 12 and shows high similarity to *OCT4A* (Fig. 1b). However, *POU5F1P3* is translated to a truncated protein with a complete N-terminal and partial POU domain because of point mutations in the *POU5F1P3* gene^6^. The POU5F1P3 protein is localized in the cytoplasm and a previous study showed that *POU5F1P3* was expressed in undifferentiated cells^16^. For instance, *POU5F1P3* was highly expressed in undifferentiated NT2 (NTERA-2) cells, a known pluripotent human embryonal carcinoma cell line, while expression of *POU5F1P3* was dramatically down-regulated upon enhancement of neural differentiation^16^. Not only undifferentiated cells, but also several cancer cells expressing *POU5F1P3* gene, may influence patient survival (Fig. 6a–i). To confirm the expression status of the *POU5F1P3* gene in cancer, we applied the Oncomine database in various cancer types with a significant *p*-value. *POU5F1P3* was highly expressed in colorectal, kidney and seminoma cancer compared to in their normal tissues, and was associated with a poor probability of patient survival (Fig. 6a–d,f,i; Supplementary Table S13). In contrast, *POU5F1P3* showed lower expression in lymphoma and breast cancer than in their normal tissues, resulting a poor survival rate of patients, and survival plots were extracted using the Kaplan Meier-plot, PrognoScan, and Gene Expression Profiling and Interactive Analyses (GEPIA) database (Fig. 6e–h; Supplementary Tables S13, S14), indicating that the expression of *POU5F1P3* is involved in the clinical outcomes of patients.

Co-expression of *POU5F1P3* was then assessed using the Oncomine and TCGA database for identifying critical signaling pathways involved in *POU5F1P3* expression (Supplementary Fig. S3). The co-expression profile of *POU5F1P3* was retrieved across 65 colorectal cancer and 65 normal sample (Supplementary Fig. S3a, left panel). Notably, *POU5F1P3* was highly correlated with *OCT4* and *POU5F1P4*. Additionally, the co-expression genes of *POU5F1P3* was extracted from TCGA database using cBioPortal, and then the extracted genes list was incorporated in the DAVID functional annotation program to determine the possible underlying signaling pathway involving *POU5F1P3* (Supplementary Fig. S3a, right panel). *POU5F1P3* is likely associated with fatty liver disease, immunodeficiency, and the sphingolipid signaling pathway in colorectal cancer, suggesting that *POU5F1P3* expression regulates the progression of colorectal cancers and their clinical outcomes through the cellular immune response, signal transmission, and liver disease outbreak.

### POU5F1P4

**Figure 7:**
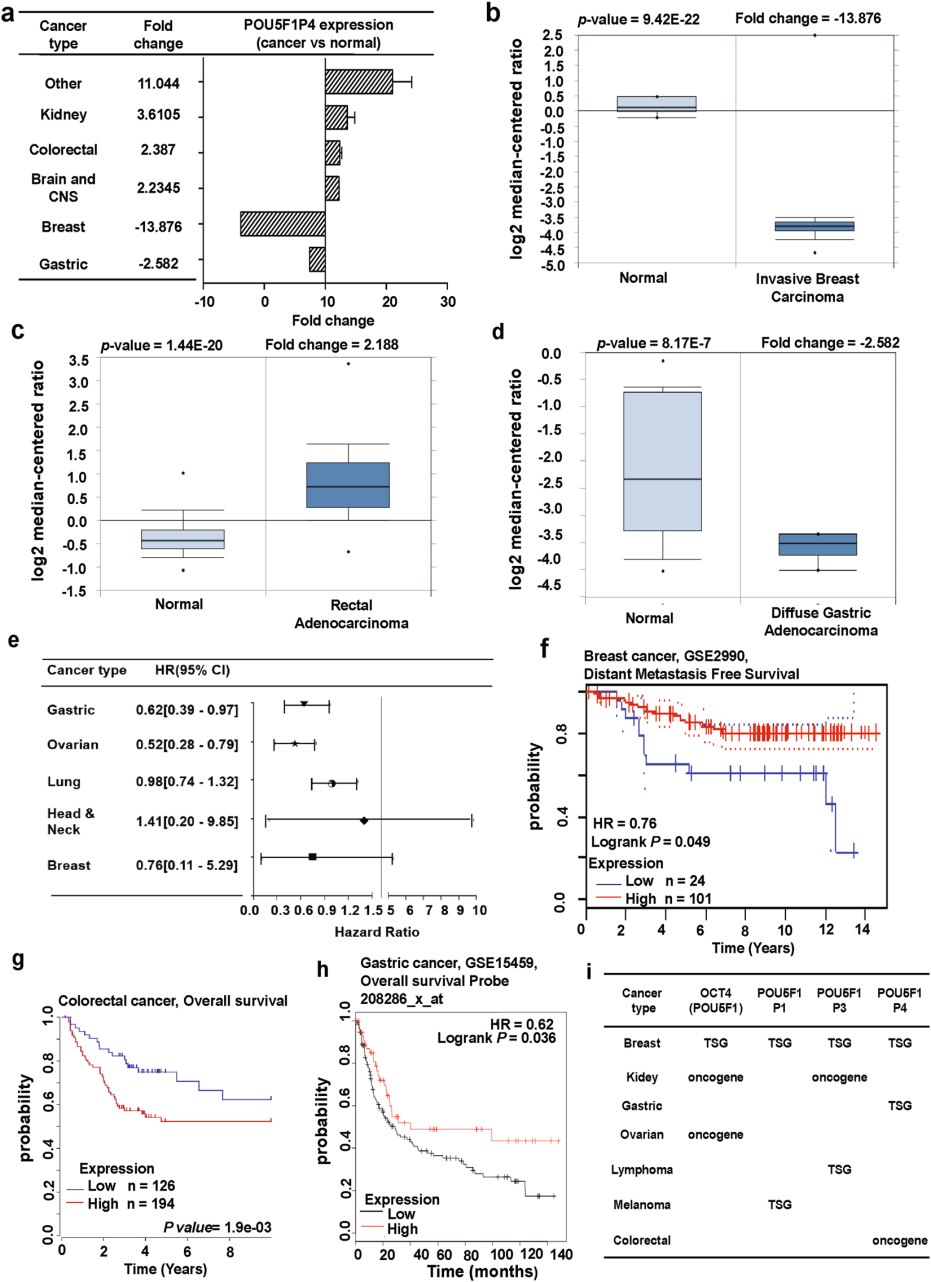
*POU5F1P4* expression pattern and patient survival analysis in different cancer types compared to *POU5F1P4* expression in normal tissue and each cancer tissue: (**a**) The fold-change of *POU5F1P4* in various types of cancers was identified from our analyses shown in Supplementary Table S15 and expressed as the forest plot. (**b–d**) The box plot comparing specific *POU5F1P4* expression in normal (left plot) and cancer tissue (right plot) was derived from the Oncomine database. The analysis was shown in breast carcinoma relative to normal breast (**b**), in rectal adenocarcinoma relative to normal rectal tissue (**c**), in gastric adenocarcinoma relative to normal gastric tissue (**d**). (**e**) Significant hazard ratios in various types of cancers were identified from our analyses shown in Supplementary Table S16 and expressed as the forest plot. (**f–h**) The survival curve comparing patients with high (red) and low (black, blue) expression in breast (**f**), colorectal (**g**), and gastric (**h**) tissue was plotted from the PrognoScan database, R2: Genomics analysis and visualization platform, and Kaplan-Meier plotter. The survival curve was analyzed with a threshold Cox *p*-value < 0.05. (**i**) The summary of predictive role of *OCT4* (*POU5F1*) and its three pseudogenes in different cancers is based on the consistent results of gene expression and outcome.

*OCT4-pg4*, also known as *POU5F1P4*, is in chromosome 1 (Fig. 1b). *POU5F1P4* is transcribed from a similar exon structure as *OCT4A* but cannot produce a stable protein. Theoretically, the POU5F1P4 protein has intact N-terminal and POU domains, but is missing a large segment of the C-terminal domain^16^. *POU5F1P4* was reported to function as a competing endogenous RNA (ceRNA) and protected *OCT4* transcription from being repressed by miR-145, which stimulated the cell growth and tumorigenicity of hepatocellular carcinoma^39,41^. Here, we aimed to confirm whether *POU5F1P4* was differentially expressed in various cancer types using the Oncomine database. The data showed that *POU5F1P4* was over-expressed in several cancer tissues including brain and CNS (central nervous system), colorectal, kidney and other cancers compared to in their normal tissues (Fig. 7a,c; Supplementary Table S15), whereas lower expression was observed in breast and gastric cancer than in their normal tissues (Fig. 7a,b,d; Supplementary Table S15). We then overviewed the prognostic value of *POU5F1P4* expression in various cancer types (Fig. 7e; Supplementary Table S16). Specifically, the results from the PrognoScan and Kaplan Meier-plot databases showed that the poor outcome of patient survival was associated with low expression of *POU5F1P4* in breast and gastric cancers (Fig. 7f,h). By contrast, the result from the R2: Genomics analysis and visualization platform showed that high expression of *POU5F1P4* was associated with a poor prognosis in colorectal cancer (Fig. 7g), further suggesting that expression of *POU5F1P4* regulates both cancer progression and clinical outcomes. Next, the Oncomine database was used to analyze the *POU5F1P4* co-expression profile. We identified the co-expression profile of *POU5F1P4* across 53 breast cancer and 6 normal tissues. As shown in the left panel of Supplementary Fig. S3b, we found that *POU5F1P4* was predominately correlated with *IDH3B* (isocitrate dehydrogenase 3 (NAD (+)) beta), *FKBP4* (FK506-binding protein 4), *YWHAE* (14-3-3 protein epsilon), and others. In addition, the correlation of *POU5F1P4* with various genes associated with colorectal cancer are shown in Supplementary Fig. S3c, left panel. Next, we retrieved the co-expression genes of *POU5F1P4* from TCGA database and analyzed these genes using the DAVID functional annotation program. The results revealed the potential signaling of *POU5F1P4* involved in breast and colorectal cancers (Supplementary Fig. S3b,c; right panel). In breast cancer, the predominant signaling pathway regulating *POU5F1P4* is the metabolic pathway, which showed the highest level of significance (Supplementary Fig. S3b, right panel). *POU5F1P4* expression may also play a role in olfactory transduction and the Wnt signaling pathway in colorectal cancer (Supplementary Fig. S3c, right panel). The function of OCT4 and its pseudogenes has not been thoroughly examined in cancer cells. In this perspective, our systematic analysis based on a variety of bioinformatics databases may help researchers determine the role of OCT4 and its pseudogenes in cancer and can be targeted as potential oncogenic markers or tumor suppressor markers for cancer treatment.

### Clinical prognosis of the co-expression of *OCT4* and its pseudogenes

**Figure 8:**
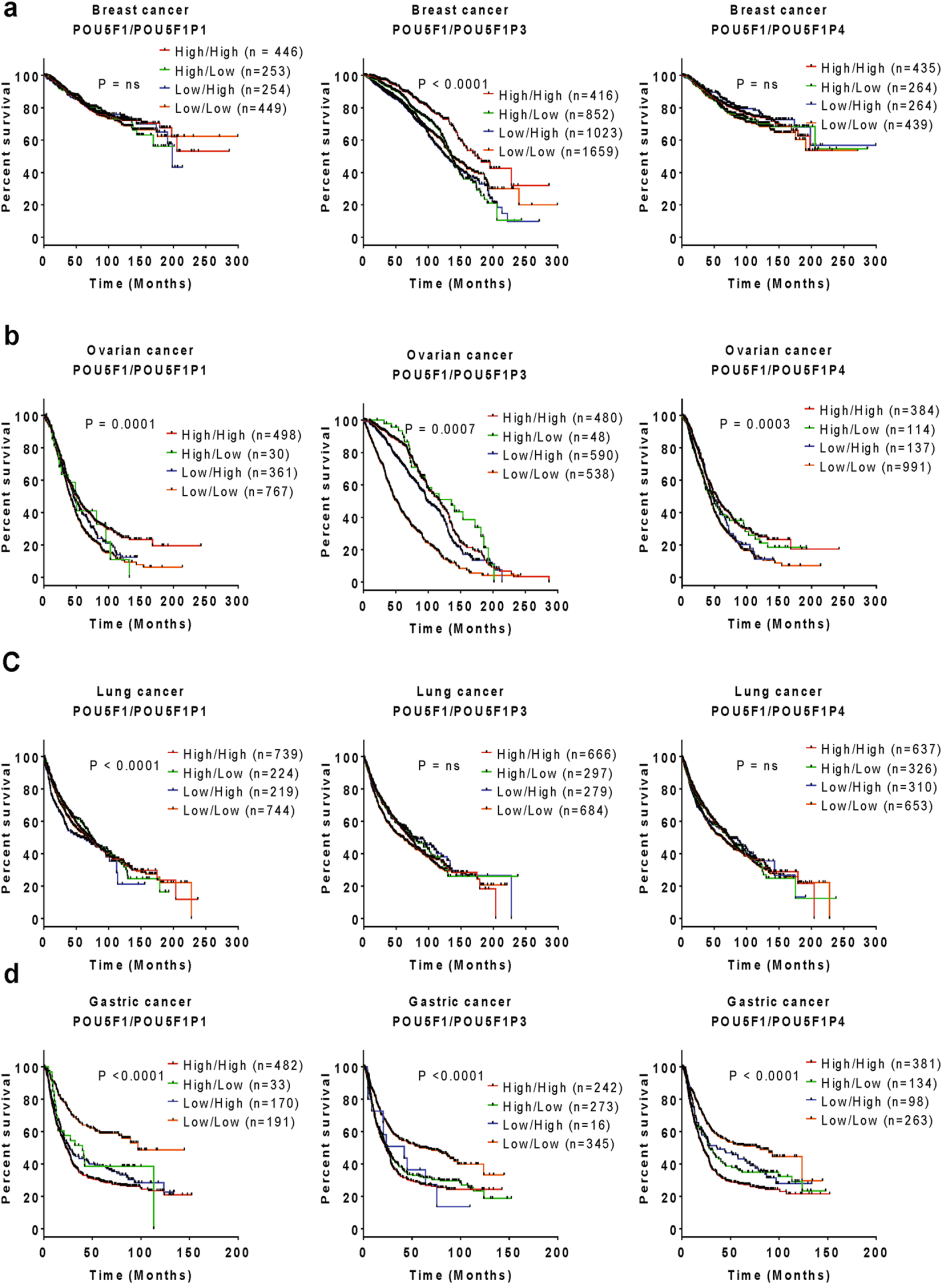
Expression co-occurrence of *OCT4* and its pseudogenes in relation to the clinical prognosis of cancer patients. The multivariate survival curves compare the clinical prognosis in patients with high/high (red), high/low (green), low/high (blue), and low/low (orange) expression co-occurrence of *POU5F1/POU5F1P1, POU5F1/POU5F1P3*, and *POU5F1/POU5F1P4* in breast (**a**), ovarian (**b**), lung (**c**), and gastric (**d**) cancers. The clinical outcome data were retrieved from the Kaplan-Meier plotter database. Information indicating statistical significance represents *p* < 0.05, and a non-significant *p*-value is expressed as ‘ns’ in the graph.

To predict the relationship between prognosis and the co-expression of *OCT4* (*POU5F1)* and its pseudogenes, we retrieved clinical prognosis data from patients with various types of cancers, including breast, ovarian, lung, and gastric cancers, using the Kaplan-Meier plotter database (Fig. 8 and Supplementary Fig. S4). The clinical prognosis data were then used to prepare a multivariate survival plot for co-occurring gene pairs, including *OCT4*/*POU5F1P1*, *OCT4*/*POU5F1P3, OCT4*/*POU5F1P4, POU5F1P1*/*POU5F1P3, POU5F1P1*/*POU5F1P4*, and *POU5F1P3*/*POU5F1P4*, with high/high, high/low, low/high, and low/low expression co-occurrence for each group. The primary endpoint for the analysis was overall survival (OS).

The results of our bioinformatics analysis showed that the expression levels of both *OCT4* and *POU5F1P1* were lower in breast cancer tissues compared to those in the normal tissue counterparts, leading to a poor prognosis (see Figs 2 and 4). Based on this expression pattern, we first performed multivariate survival analysis of *OCT4/POU5F1P1* co-expression in breast cancer; however, we found a non-significant survival probability among the high/high, high/low, low/high, and low/low variable groups for this gene pair (Fig. 8a, left panel). Similar to *OCT4* and *POU5F1P1*, the expression level of *POU5F1P3* was also low in breast cancer tissues, which was associated with poor patient survival (see Fig. 7). Interestingly, the multivariate survival analysis revealed a significantly poor prognosis when *OCT4/POU5F1P3* expression was high/low or low/high compared to the high/high or low/low group (Fig. 8a, middle panel), suggesting that partial-co-occurrence of *OCT4/POU5F1P3* may regulate cancer prognosis. *OCT4/POU5F1P4* co-expression had a non-significant effect on clinical prognosis (Fig. 8a, right panel). With respect to the co-expression patterns of *POU5F1P1*/*POU5F1P3, POU5F1P1*/*POU5F1P4*, and *POU5F1P3*/*POU5F1P4* in breast cancer tissue, the survival probability was significantly poor in the low/low group of *POU5F1P3*/*POU5F1P4*, whereas the other co-occurrence groups showed non-significant survival probabilities (Supplementary Fig. S4a). These results indicate that partial-/non-co-occurrence of *OCT4* with its three pseudogenes (*POU5F1P1*, *POU5F1P3*, and *POU5F1P4*) may regulate clinical outcomes in breast cancer patients.

In the case of ovarian cancer, the partial-co-occurrence of *OCT4/POU5F1P1* (high/low) was associated with a poorer prognosis compared to that for the high/high, low/high, and low/low groups (Fig. 8b, left panel). By contrast, high expression levels of both *OCT4* and *POU5F1P3* were associated with poor survival outcomes in patients with ovarian cancer (Fig. 8b, middle panel). These results imply that *POU5F1P3* may interact with *OCT4* and regulate tumor progression in ovarian cancer. Moreover, patients with low/low or low/high co-expression of *OCT4/POU5F1P4* appeared to have significantly poorer survival than that of the other groups of ovarian cancer patients (Fig. 8b, right panel). The patients with non-co-occurrence of *POU5F1P1*/*POU5F1P3, POU5F1P1*/*POU5F1P4*, or *POU5F1P3*/*POU5F1P4* showed a significantly poorer prognosis than that of the full or partial co-occurrence groups (see Supplementary Fig. S4b). In lung cancer, a poor prognosis was observed in the group of patients showing a low/low co-expression pattern of *OCT4*/*POU5F1P1* compared to the other groups (high/low, low/high, or high/high *OCT4*/*POU5F1P1* expression) (Fig. 8c, left panel). However, the survival pattern of patients with *OCT4/POU5F1P3, OCT4/POU5F1P4, POU5F1P1*/*POU5F1P3, POU5F1P1*/*POU5F1P4*, or *POU5F1P3*/*POU5F1P4* co-occurrence was not statistically significant (Fig. 8c, middle and right panel; Supplementary Fig. S4c).

We next focused on gastric cancer. The clinical outcomes of patients with partial *OCT4/POU5F1P1* co-occurrence (high/low) were significantly poorer than those of patients with non *OCT4/POU5F1P1* co-occurrence, and a co-occurrence (high/high) and partial co-occurrence pattern (low/high) of *OCT4/POU5F1P1* was also related with a poorer prognosis compared to that of the non-co-occurrence group (Fig. 8d, left panel). This finding suggests that co-occurrence or partial co-occurrence of *OCT4/POU5F1P1* may regulate the clinical outcomes in gastric cancer patients. Similar survival patterns were observed for gastric cancer patients with *OCT4/POU5F1P3*, *OCT4/POU5F1P4, POU5F1P1*/*POU5F1P3, POU5F1P1*/*POU5F1P4*, or *POU5F1P3*/*POU5F1P4* co-occurrence (see Fig. 8d, middle and right panels and Supplementary Fig. S4d).

Thus, our multivariate survival analyses revealed that the co-occurrence/partial-/non-co-occurrence of *OCT4* and its three pseudogenes modulated the clinical outcomes for patients with certain types of cancers, which might open a new door toward elucidating the underlying mechanism of cancer prognosis regarding the expression of *OCT4* and its three pseudogenes. Furthermore, the interactions among *OCT4*, *POU5F1P1*, *POU5F1P3*, and *POU5F1P4* might be associated with the progression of various types of cancers.

## Discussion

*OCT4* (*POU5F1*) is commonly expressed in induced pluripotent stem and stem cells and is an important factor for maintaining pluripotency and stemness^10^. However, it has also been reported that OCT4 may participate in the maintenance of stemness in somatic cancer tissues (bladder^42^, squamous cell carcinoma^43^, and breast carcinoma^44,45^) and be involved in the proliferation and metastasis of several cancer cells^10–12^. *OCT4* can produce three transcript variants (*OCT4A*, *OCT4B*, *OCT4B1*) by alternative splicing. Among its three transcript variants, *OCT4A* has been shown to maintain the pluripotency and self-renewal of iPSCs and ESCs^9^. *OCT4A* is not only expressed in normal stem cells, but is also undoubtedly expressed at various levels in a variety of human cancer cell lines^46^. Zhou *et al*. also provided clear evidence that *OCT4A* is expressed at both the mRNA and protein levels in somatic cell carcinoma cells^47^. In addition, they revealed that knockdown of *OCT4A* in somatic cell carcinoma cells resulted in a reduction of the c-FOS (encoded by the cellular oncogene *c-FOS*) protein level, led to aberrant activator protein 1 (AP-1) signaling, dampened the self-renewal capacity of the cells, and caused deficient cell migration related to cell growth retardation^47^. In contrast to *OCT4A* maintaining the stemness of stem cells and affecting the characteristics of cancer cells, two other *OCT4* transcript variants (*OCT4B* and *OCT4B1*) do not appear to be involved in maintaining stemness. In fact, each variant of *OCT4* shows differential expression patterns in cancer and somatic cells and thus may have differential functions^23,48–52^. However, available qPCR or RT-PCR primers are generally not able to distinguish between *OCT4* isoforms; thus, our bioinformatics analyses with the current literature and databases may have limitations and certain flaws. With the recent development of true *OCT4* isoform-specific primers^46,47^, it will now be possible to overcome this limitation in future studies.

Several known pseudogenes of *OCT4* have been reported to regulate gene function. Among them, three are known to be expressed in various cancers or cancer stem cells, iPSCs, and ESCs^13,16^. Although OCT4 and its pseudogenes have been identified as transcription factor for maintaining iPSCs and ESCs or are involved in cancer development, it remains unclear whether they act as oncogenes or tumor suppressors in cancer progression and prognosis. Thus, we systematically analyzed the expression patterns of *OCT4* and its pseudogenes and determined the correlation between expression and clinical outcomes in various cancer types. By using the Oncomine database, differential expression patterns were observed depending on the cancer cell type (Supplementary Table S1). Furthermore, analysis from diverse databases showed that differential *OCT4* expression patterns are related to the patient survival ratio (Fig. 2f–h; Supplementary Table S2), which agrees with the results of previous studies^10,11,53^. For example, *OCT4* expression protected against metastasis of breast cancer cells but increased tumorigenesis in cervical cancer cells^10,53^. The results of these previous studies showed that *OCT4* expression in breast cancer cells reduces metastasis, which is similar to our results from analyzing several databases (Fig. 2a,b). Kaplan-Meier plot analysis also demonstrated that high *OCT4* expression was associated with better patient survival (Fig. 2f). Additionally, our systematic data showed that pseudogenes, which were previously considered as junk DNA, are expressed in cancer cells and can affect cancer cell characteristics. According to large-scale genome-wide studies, *POU5F1P1* is expressed in various cancer tissues including breast colorectal, ovarian, and prostate tumors^19,54–56^. Moreover, a previous study reported that *POU5F1P1*, a known *OCT4* pseudogene, was located 15 kbp downstream of the SNP (single-nucleotide polymorphism) rs6983267 and is strongly correlated with an increased risk of colon and prostate cancer^14^. It was also reported that POU5F1P1 shows 95% homology to the OCT4 protein and can trigger ectopic expression of reporter genes in HeLa cells^14^. Another report identified *POU5F1P3* genes that are differentially expressed in normal, testicular cancer, and testicular tumor progression stage (cancer) tissue samples^57^ and suggested that the *POU5F1P1* and *POU5F1P3* genes are the causative genes of various cancers. We obtained similar results through bioinformatics analysis, which demonstrated that expression of *POU5F1P1* and *POU5F1P3* regulated cancer progression and clinical outcomes of patients (see Figs 4–6).

It was also reported that POU5F1P4, another pseudogene of *OCT4*, regulates the expression of *OCT4*^32,34^. Therefore, POU5F1P4 may influence cancer development by regulating *OCT4* expression. Recently, a study demonstrated that expression of *POU5F1P3* and *POU5F1P4* was detected and potentially increased in urothelial cancer compared to in normal urothelial cells^58^. The expression of pseudogenes (*POU5F1P1*, *POU5F1P3*, and *POU5F1P4*) in numerous solid tumors has been described, but little is known about the function of these pseudogenes. A previous study demonstrated that *POU5F1P1*, *POU5F1P3*, and *POU5F1P4* were expressed in human tumors, but the effects on transcription or cell growth were not measurable^40^. The results of our systematic analysis agree with studies showing that *OCT4* and its pseudogenes (*POU5F1P1*, *POU5F1P3*, and *POU5F1P4*) are expressed in various cancer cells and affected patient survival. Moreover, multivariate survival analysis revealed a correlation between the co-expression of *OCT4* and its pseudogenes with clinical outcomes of patients with certain cancer types, suggesting a link between survival and *OCT4* and its pseudogenes co-occurrence. In other words, the differential expression and function of *OCT4* and its pseudogenes may depend on the type of cancer cells. Experimental results for *OCT4* and its pseudogene expression revealed their involvement in the development of various cancers and effects on clinical outcomes, but the underlying mechanism remains unknown.

In this systematic study we provided some evidence regarding the relationship between expression alterations of *OCT4* and its pseudogenes and clinical outcomes and improved the understanding of large-scale genome-wide oncogenic data, which may facilitate translation of this genomic knowledge into clinical practice. Thus, our analysis may provide a foundation for determining the function of OCT4 and its pseudogenes in various cancer cells.

## Conclusion

In this study, we provided detailed information regarding the expression of *OCT4* and its pseudogenes and the correlation of this expression with clinical prognosis in various cancer types. Results of our systematic analysis showed that *OCT4* and its pseudogenes were differentially expressed in cancers and associated with the clinical outcomes of patients. We provide an overview of the role of *OCT4* and its three pseudogenes and whether they function as oncogenes or tumor suppressor genes. According to our systematic analysis, *OCT4* most likely functions as a tumor suppressor gene in breast cancer, but appears to function as an oncogene in kidney and ovarian cancers. Similarly, the three *OCT4* pseudogenes normally function as tumor suppressor genes in various cancer types, while *POU5F1P3* in kidney cancer and *POU5F1P4* in colorectal cancer may function as oncogenes (see Fig. 7i). We also demonstrated differences in the multivariate patient survival patterns according to the expression correlation between *OCT4* and its pseudogenes, revealing that the co-occurrence of *OCT4* and its three pseudogenes in various cancers can differentially regulate tumor progression as well as prognosis. Collectively, these results suggest that activators or inhibitors can be designed to target cancers expressing *OCT4* and its pseudogenes and may be useful for cancer and cancer stem cell treatment.

## Material and Methods

### Oncomine database analysis

The expression level of *OCT4* and its pseudogenes in various cancer types was retrieved from the Oncomine database (https://www.oncomine.org/resource/login.html)^59,60^. The fold-change of mRNA expression in cancer tissue compared to in their normal counterparts was acquired using parameters of a threshold *p*-value of 1E-4; fold-change of 2; and gene ranking in the top 10%; the precise analyses are summarized in Table 1, Supplementary Tables S1, S7, S13, and S15, respectively. The co-expression profiles of *OCT4* and its pseudogenes in different cancer types were extracted and illustrated as a heat map.

**Table 1:** Main characteristic of the selected oncogenomic portals.

Database	Data source	Sites of analyzed cancer*	Oncogenomic data	link
Oncomine	TCGA, Cancer data from literature	Bd; Br; Bra; Cer; Clr; Eso; HN; Kd; Lng; Lvr; Lymph; Ov; Pnc; also: cancer cell lines	Drug sensitivity, cancer histology, clinical outcome, tissue, pathology, subtype, molecular subtype, patient treatment response	https://www.oncomine.org^59,60^
PrognoScan	Cancer data from literature	Bd; Bld; Br; Bra; Clr; EA; Eso; HN; Kd; Lng; Lymph; Ov; Prst; Sk; ST;	Survival analyses	http://www.abren.net/PrognoScan^62^
STRING	Protein, gene from literature	Gene, gene from literature	Structure	http://stringdb.org^66,68^
cBioPortal	AMC, BCCRC, BGI, British Columbia, Broad, Broad/Cornell, CCLE, CLCGP, Genentech, ICGC, JHU, Michigan, MKSCC, MKSCC/Broad, NCCS, NUS, PCGP, Pfizer UHK, Riken, Sanger, Singapore, TCGA, TSP, UTokyo, Yale	ACC; Bd; Bld; Br; Bra; Chl; Clr; Eso; HN; Kd; Lng; Lvr; Lymph; MM` Npx; Ov; Pnc; Prst; Sk; ST; Stc; Thr; Utr; also: cancer cell lines	Mutations, putative copy number alterations; mRNA expression, protein/phosphoprotein level; survival analyses	http://www.cbioportal.org/^67,69^
DAVID functional annotation	—	Signal pathway	GO terms, annotation terms, BioCarta & KEGG pathway, interacting proteins, gene-disease associations, protein functional domains and motifs	https://david.ncifcrf.gov/home.jsp^70^
OncoLnc	TCGA	Bld: Br: Cer: Col: Eso: Gil: Head; Kd; Leuk; Bra; Lvr; Lng; Ov; Panc; Reect; Src; Stm; MM;	Survival analyses	http://www.oncoLnc.org/^63^
PROGgeneV2	TCGA, Cancer data from literature	Br; Kd; Bld; Bon; Bra; Col; Heme; Hnc; Liv; Lng; Ov; Panc; Prs; Rect; Skn; Stm; Uter; Cerv; Eso; Eye; Gst; Mstl; Nure; Src; Tym; Tyrd; also: cancer cell lines	Survival analyses	http://watson.compbio.iupui.edu/chirayu/proggene/database/index.php^65^
Kaplan-Meier plotter	GEO (Affymetrix microarrays only), EGA and TCGA	Br; Gst; Ov; Lng; Liv;	Survival analyses	http://kmplot.com/analysis/^61^
GEPIA	RNA sequencing expression data from TCGA and the GTEx project	Acc; Blca; Brca; Cesc; Chol; Coad; Dlbc; Esca; Gbm; Hnsc; Kich; Kirc; Kirp; Laml; Lgg; Lihc; Luad; Lusc; Meso; Ov; Paad; Pcpg; Prad; Read; Sarc; Skcm; Stad; Tgct; Thca; Thym; Ucec; Uvm	Survival analysis, Methylation, Annotation, WGS, SNP, Chip, CGHt	http://gepia.cancer-pku.cn/index.html^71^
R2: Genomics analysis and visualization platform	GEO, TCGA, and GTEx projects	Gli; Kicc; Lug; Lym; Mlym; Mal; Myel; Neur; Ova; Pan; Wil	Survival analysis	https://hgserver1.amc.nl/cgi-bin/r2/main.cgi?&species=hs^72^

^*^Abbreviations: ACC– adenoid cystic carcinoma; Bd – bladder; Bld – blood; Bo – bone; Br – breast; Bra – brain; Chl – cholangiocarcinoma; Clr – colorectal; Col – colon; EA – eye and adnexa; EG - endocrine glands; Eso – esophagus; GIST – gastrointestinal; HN– head and neck; Htp – hematopoietic; Kd – kidney; Lng – lung; Lvr – liver and biliary tract; Lymph – Lymphoma; Msh –mesothelioma; Mth – mouth; Nb – neuroblastoma; Npx – nasopharynx; Ov – ovary; Pan – pancancer; Pnc – pancreas; Pnx– pharynx; Prc/Prn - pheochromocytoma and paraganglioma; Prst – prostate; Rc – rectum; Sk – skin; ST – soft tissues; Stc– stomach; Swn – schwannoma; Thm – thymus; Thr – thyroid; Tst – testis; Utr – uterine; Blca – bladder urothelial carcinoma; Brca – breast invasive carcinoma; Cesc – cervical squamous cell carcinoma and endocervical adenocarcinoma; Chol – cholangio carcinoma; Coad – colon adenocarcinoma; Dlbc – lymphoid neoplasm diffuse large B-cell lymphoma; Esca –esophageal carcinoma; Gbm – glioblastoma multiforme; Hnsc – head and neck squamous cell carcinoma; Kich – kidney chromophobe; Kirc – kidney renal clear cell carcinoma; Kirp – kidney renal papillary cell carcinoma; Laml – acute myeloid leukemia; Lgg – brain lower grade glioma; Lihc – liver hepatocellular carcinoma; Luad – lung adenocarcinoma; Lusc – lung squamous cell carcinoma; Meso – mesothelioma; Ov – ovarian serous cystadenocarcinoma; Paad – pancreatic adenocarcinoma; Pcpg – pheochromocytoma and paraganglioma; Prad – prostate adenocarcinoma; Read – rectum adenocarcinoma; Sarc – sarcoma; Skcm – skin cutaneous melanoma; Stad – stomach adenocarcinoma; Tgct – testicular germ cell tumor; Thca – thyroid carcinoma; Thym – thymoma; Ucec – uterine carcinoma; Uvm – uveal melanoma; Myel – myeloma; Neur – neuroblastoma; Wil – Wilms.

### Kaplan-Meier plotter database analysis

The Kaplan-Meier plotter can be used to evaluate the effect of 54,675 genes on patient survival using 10,461 cancer samples (5,143 breast, 1,816 ovarian, 2,437 lung, and 1,065 gastric cancer) with the HGU133 Plus 2.0 array. The correlation between the expression of *OCT4* and its pseudogenes and survival in breast, gastric, lung, and ovarian was analyzed by Kaplan-Meier plotter (http://kmplot.com/analysis/)^61^. The log rank *p*-value and hazard ratio with 95% confidence intervals were also calculated.

### PrognoScan database analysis

The correlation between the expression of *OCT4* and its pseudogenes and survival in various cancer types was also investigated using the PrognoScan database (http://www.abren.net/Progno-Scan/)^62^. The significant threshold was adjusted to a Cox *p*-value < 0.05 and the results are summarized in Supplementary Tables S2, S8, S14 and S16.

### OncoLnc database analysis

The OncoLnc (http://www.oncolnc.org/) is a tool for interactively investigating survival correlations and retrieving clinical data linked to expression data for mRNAs, miRNAs, or long non-coding RNAs^63^. OncoLnc contains survival data for 8,647 patients from 21 cancer studies implemented by The Cancer Genome Atlas (TCGA), along with RNA-seq expression data for mRNAs and miRNAs from TCGA and long non-coding RNA expression from MiTranscriptome beta. The OncoLnc provides data for the Cox analysis for up to 21 cancers related to *OCT4* and its pseudogenes.

### PROGgeneV2 database analysis

PROGgeneV2 was used to confirm the relationship between the expression of *OCT4* and its pseudogenes and prognostic outcomes in various cancer types (http://watson.compbio.-iupui.edu/chirayu/proggene/database/index.php). PROGgeneV2 contains data from 134 cohorts from 21 cancer types^64^. Only data with significant *p*-values were selected for analysis (*p*-value < 0.05).

### Identifying protein components of OCT4 and its pseudogenes axis

The STRING analysis tool was performed to predict interacting proteins using human OCT4 and its pseudogenes as queries (http://stringdb.org). Numerous known partners have been genetically confirmed and therefore provided a foundation for identifying other protein partners in the axis. Any proteins identified that were not specific to the OCT4 and its pseudogenes axis were disqualified from the gene signature^65,66^.

### cBioPortal database analysis

We conducted an integrative analysis of *OCT4* and its pseudogenes and clinical characteristics using cBioPortal data, an open access resource at http://www.cbioportal.org/ ^67,68^, which currently provides access to data from more than 48,668 tumor samples from 172 cancer studies in the TCGA pipeline. The query interface combined with customized data storage enabled us to interactively explore genetic alterations across samples curated from national and international cancer studies and specific genes. The primary search parameters included alterations (amplification, deep deletion, missense mutations), CNA from GISTIC, and RNA-seq data with the default setting. For the secondary search, we focused on RNA-seq data.

### DAVID (6.8) functional annotation database analysis

The Database for Annotation, Visualization and Integrated Discovery (DAVID) v6.8 (https://david.ncifcrf.gov/) comprises a full knowledgebase of web-accessible programs which now provides a comprehensive set of functional annotation tools for investigators to understand the biological meaning behind biological processes. We retrieved genes co-expressed with *OCT4* and its pseudogenes from cBioPortal and analyzed these genes using DAVID to explore predicted signaling pathways. We ranked the pathways according to their significant *p*-values. We selected top 10 biological pathways with significant *p*-values in each cancer and graphed the results (*p*-value < 0.05).

### GEPIA database analysis

GEPIA (http://gepia.cancer-pku.cn/index.html) is a web server for investigating the RNA sequencing expression, co-expression, and survival data of 8,587 normal and 9,736 tumors samples from TCGA and the GTEx project. In this study, we performed survival analysis for the expression of *OCT4* and its pseudogenes in breast cancer using GEPIA online tools.

### R2: Genomics analysis and visualization platform database analysis

The R2 platform (https://hgserver1.amc.nl/cgi-bin/r2/main.cgi?&species=hs) is a web server for investigating the RNA sequencing and microarray data of expression, co-expression, and survival data of normal and tumor samples from the TCGA, GEO, and GTEx projects. In this study, we performed survival analysis for the expression of *OCT4* and its pseudogenes in breast cancer using R2 online tools.

### Statistical analysis

The bar and forest plot were drown using GraphPad Prism version 7 (GraphPad Software, La Jolla, CA, USA). Survival curves were extracted from the PrognoScan, PROGgeneV2, OncoLnc, and Kaplan-Meier plotters. All results are displayed with *p-*values obtained from the log-rank test. Similarly, using Oncomine and heat maps, the significance of the data (*P*-values) was determined by the program. The multivariate survival analysis was performed by GraphPad Prism version 7 software using data retrieved from the Kaplan-Meier plotter database; the significance of the data (Log-rank Mantel-Cox *p*-values) was calculated by the software.

## Electronic supplementary material

Supplementary information
