# Supplementary material for: Systematic expression alteration analysis of master reprogramming factor OCT4 and its three pseudogenes in human cancer and their prognostic outcomes
Source: Sci Rep. 2018 Oct 4;8:14806. doi: 10.1038/s41598-018-33094-7 (PMC6172215; doi:10.1038/s41598-018-33094-7)
Supplement: Supplementary file 1 — Supplementary information [file 41598_2018_33094_MOESM1_ESM.pdf]

Systematic expression alteration analysis of master reprogramming factor OCT4 and its three pseudogenes in human cancer and their prognostic outcomes

Subbroto Kumar Saha†, Yeojin jeong†, Sungha Cho, and Ssang-Goo Cho*

Department of Stem Cell and Regenerative Biotechnology, Incurable Disease Animal Model & Stem Cell Institute (IDASI), Konkuk University, Seoul 05029, Republic of Korea; subbroto@konkuk.ac.kr (S.K.S.); jyj05787@gmail.com (Y.J.); shgool@naver.com (S.C.)

* Correspondence: ssangoo@konkuk.ac.kr; Tel: +82 2-450-4207

† These authors contributed equally to this work.

Supplementary Tables

Supplementary Table S1. *OCT4 (POU5F1)* expression in each Cancer types type.

Cancer types	Cancer subtypes	*p*-value	Fold-change	Rank (%)	Sample	Reference
Bladder	Superficial Bladder Cancer types	1.97E-8	5.279	1	54	[1]
	Superficial Bladder Cancer types	1.08E-11	4.969	9	157	[2]
	Infiltrating Bladder Urothelial Carcinoma	5.79E-6	2.067	11	157	[2]
Breast	Invasive Breast Carcinoma	4.12E-29	-5.513	2	59	[3]
	Invasive Ductal Breast Carcinoma	6.29E-4	-1.831	4	66	[4]
	Ductal Breast Carcinoma in Situ	3.10E-4	-1.769	4	66	[4]
Colorectal	Rectal Adenocarcinoma	3.17E-22	2.514	4	130	[5]
	Rectal Adenocarcinoma	9.79E-6	1.424	2	105	[6]
	Cecum Adenocarcinoma	3.39E-8	1.567	2	105	[6]
	Rectosigmoid Adenocarcinoma	2.55E-6	1.591	2	105	[6]
Kidney	Clear Cell Renal Cell Carcinoma	3.95E-5	2.941	7	20	[7]
	Papillary Renal Cell Carcinoma	4.86E-6	1.426	12	92	[8]
Other	Seminoma, NOS	1.20E-10	11.699	1	107	[9]
	Mixed Germ Cell Tumor, NOS	1.24E-9	4.005	5	107	[9]
	Embryonal Carcinoma, NOS	4.93E-7	7.100	7	107	[9]
	Testicular Embryonal Carcinoma	9.66E-4	11.157	2	30	[10]
Ovarian	Ovarian Serous Adenocarcinoma	7.46E-10	6.313	2	53	[11]
	Ovarian Clear Cell Adenocarcinoma	7.10E-6	1.799	3	103	[12]
	Ovarian Endometrioid Adenocarcinoma	6.99E-7	1.388	5	103	[12]
	Ovarian Serous Adenocarcinoma	2.16E-5	1.289	8	103	[12]
Sarcoma	Fibrosarcoma	5.78E-5	-4.038	1	53	[13]
	Leiomyosarcoma	1.57E-4	-1.187	11	158	[14]

***Abbreviations:** NOS **-** Not Otherwise Specified

Supplementary Table S2. Association of *OCT4* expression and survival of Cancer types patients (PrognoScan).

**Cancer types**	**N**	**Cox *p*-value**	**HR**	**Endpoint**	**Dataset**	**Probe ID**
Lung	117	0.001101	0.6	Overall Survival	GSE13213	A_23_P59138
	117	0.002718	0.67	Overall Survival	GSE13213	A_24_P144601
	117	0.005945	0.71	Overall Survival	GSE13213	A_24_P214841
	117	0.014269	0.68	Overall Survival	GSE13213	A_32_P132563
	86	0.021249	0.49	Overall Survival	MICHIGAN-LC	Z11899_s_at
Ovarian	110	0.016227	0.84	Overall Survival	GSE17260	A_24_P214841
	110	0.024797	0.82	Progression Free Survival	GSE17260	A_24_P214841
	185	0.026745	0.61	Disease Free Survival	GSE26712	208286_x_at
	110	0.027991	0.82	Progression Free Survival	GSE17260	A_24_P144601
	110	0.041767	0.85	Progression Free Survival	GSE17260	A_23_P59138
	110	0.048337	0.83	Progression Free Survival	GSE17260	A_32_P132563
	110	0.033365	0.85	Overall Survival	GSE17260	A_24_P144601
Blood	53	0.042983	1.29	Event Free Survival	E-TABM-346	208286_x_at
	53	0.04875	1.37	Overall Survival	E-TABM-346	208286_x_at
Colorectal	55	0.04826	0.16	Overall Survival	GSE17537	208286_x_at
Breast	77	0.020204	3.18	Relapse Free Survival	GSE9195	208286B1:H17_x_at

Supplementary Table S3. Alteration frequency of a five-gene signature (*POU5F1*, *SOX2*, *NANOG*, *LIN28A*, *KLF4*) in Cancer typess.

Cancer types	Data source	N	Frequency (%)	Amplification % (N)	Deletion % (N)	Mutation % (N)	Multiple alterations % (N)
Lung squ.	TCGA	178	57.30	55.06 (1)	-	0.56 (1)	1.69 (3)
Ovarian	TCGA	316	36.71	35.76 (113)	0.63 (2)	0.32 (1)	-
NEP	Trento/Comell/ Broad 2016	107	31.78	-	-	0.93 (1)	-
Esophagus	TCGA	184	29.35	27.17 (50)	1.63 (3)	0.54 (1)	-
Head & Neck	TCGA	504	25.60	23.41 (118)	0.4 (2)	1.59 (8)	0.2 (1)
NSCLC	MSK, JCO 2018	240	25.00	20.19 (231)	1.31 (15)	2.8 (32)	0.7 (8)
Testicular germ cell	TCGA	149	22.82	20.81 (31)	-	2.01 (3)	-
Cervical	TCGA	191	21.99	17.28 (33)	1.05 (2)	3.14 (6)	0.52 (1)
Pancreas	UTSW	109	20.18	9.17 (10)	9.17 (10)	-	1.83 (2)

***Abbreviations:** Lung squ.- Lung Squamous Cell Carcinoma ; NEP - Neuroendocrine Prostate ; NSCLC - Non-Small Cell Lung Cancer types -

Supplementary Table S4. Alteration frequency of a seven-gene signature (*POU5F1*, *SOX2*, *NANOG*, *LIN28A*, *KLF4*, *SALL4*, *FGF2*) in Cancer typess.

Cancer types	Data source	N	Frequency (%)	Amplification % (N)	Deletion % (N)	Mutation % (N)	Multiple alterations % (N)
Lung squ	TCGA	178	60.67	54.49 (97)	-	2.81 (5)	3.37 (6)
Ovarian	TCGA	316	41.77	37.66 (119)	1.9 (6)	0.63 (2)	1.58 (5)
NEP	Trento/Comelll/ Broad 2016	107	38.32	37.38 (40)	-	0.93 (1)	-
Esophagus	TCGA	184	34.78	30.98 (57)	2.17 (4)	1.63 (3)	-
Head & Neck	TCGA	504	26.79	23.02 (116)	0.79 (4)	2.38 (12)	0.6 (3)
NSCLC	MSK, JCO 2018	240	29.02	20.89 (239)	1.31 (15)	5.33 (61)	1.49 (17)
Stomach/Esophageal	TCGA	265	28.30	23.02 (61)	3.4 (9)	1.13 (3)	0.75 (2)
Stomach	TCGA	393	26.21	15.27 (60)	2.54 (10)	7.12 (28)	1.27 (5)
Pancreas	UTSW	109	25.69	11.01 (12)	11.01 (12)	-	3.67 (4)
Cervical	TCGA	191	25.13	17.8 (34)	1.57 (3)	4.71 (9)	1.05 (2)
Testicular germ cell	TCGA	149	23.49	20.81 (31)	-	2.68 (4)	-
The MBC project	Provisional, October 2017	103	21.36	14.56 (15)	2.91 (3)	2.91 (3)	0.97 (1)
Melanoma	TCGA	287	21.25	6.62 (19)	0.7 (2)	11.5 (33)	2.44 (7)

***Abbreviations:** Lung squ.- Lung Squamous Cell Carcinoma ; NEP - Neuroendocrine Prostate ; NSCLC - Non-Small Cell Lung Cancer types ; The MBC project – The Metastatic Breast Cancer types Project.

Supplementary Table S5. Percentages of alterations in *POU5F1*, *SOX2*, *NANOG*, *KLF4*, and *LIN28a*.

**Cancer types**	***POU5F1***	***SOX2***	***NANOG***	***KLF4***	***LIN28a***
Lung squ	0.6%	54.0%	8.0%	0.6%	0.0%
Ovarian	6.0%	26.0%	9.0%	0.7%	1.7%
NEP	19.0%	29.0%	14.0%	9.0%	29.0%
Esophagus	5.0%	3.0%	1.6%	1.6%	1.1%
Head & Neck	1.2%	21.0%	1.8%	1.8%	0.8%
NSCLC	1.4%	20.0%	3.0%	1.1%	1.2%
Testicular germ cell	0.7%	1.3%	21.0%	0.0%	0.0%
Cervical	2.1%	18.0%	0.5%	1.6%	0.5%
Pancreas	9.0%	7.0%	4.0%	0.9%	8.0%

Supplementary Table S6. Percentages of alterations in *POU5F1*, *SOX2*, *NANOG*, *KLF4*, *LIN28a*, *SALL4*, and *FGF2*.

**Cancer types**	***POU5F1***	***SOX2***	***NANOG***	***KLF4***	***LIN28a***	***SALL4***	***FGF2***
Lung squ	0.6%	54.0%	8.0%	0.6%	0.0%	4.0%	1.7%
Ovarian	6.0%	26.0%	9.0%	0.7%	1.7%	8.0%	1.9%
NEP	19.0%	29.0%	14.0%	9.0%	29.0%	27.0%	10.0%
Esophagus	5.0%	23.0%	1.6%	1.6%	1.1%	8.0%	0.5%
Head & Neck	1.2%	21.0%	1.8%	1.8%	0.8%	1.2%	0.8%
NSCLC	1.4%	20.0%	3.0%	1.1%	1.2%	5.0%	0.3%
Stomach/esophageal	6.0%	7.0%	2.6%	80.0%	1.5%	13.0%	1.9%
Stomach	4.0%	7.0%	2.3%	3.0%	1.5%	13.0%	1.5%
Pancreas	9.0%	7.0%	4.0%	0.9%	8.0%	7.0%	5.0%
Cervical	2.1%	18.0%	0.5%	1.6%	0.5%	3.0%	1.6%
Testicular germ cell	7.0%	1.3%	21.0%	0.0%	0.0%	0.7%	0.0%
The MBC project	1.3%	2.6%	6.0%	5.0%	1.3%	10.0%	0%
Melanoma	7.0%	1.0%	3.0%	1.7%	2.1%	9.0%	1.4%

Supplementary Table S7. *POU5F1P1* expression in each Cancer types.

Cancer types	Cancer subtype	*p*-value	Fold change	Rank (%)	Sample	Reference
Breast	Invasive Ductal Breast Carcinoma	6.29E-04	-1.832	4	66	[4]
Kidney	clear cell renal cell carcinoma	5.21E-06	3.752	3	67	[11]
	Papillary Renal Cell Carcinoma	7.24E-04	2.27	6	67	[11]
	Clear Cell Renal Cell Carcinoma	5.09E-05	2.302	7	20	[7]
	Hereditary Clear Cell Renal Cell Carcinoma	1.99E-05	1.575	15	70	[15]
Melanoma	Cutaneous Melanoma	4.97E-09	-3.91	3	70	[16]
	Benign Melanocytic Skin Nevus	3.31E-04	-1.964	7	70	[16]
Other	Seminoma, NOS	1.26E-12	10.539	1	107	[9]
	Mixed Germ Cell Tumor, NOS	1.36E-10	4.308	4	107	[9]
	Embryonal Carcinoma, NOS	4.73E-07	7.348	7	107	[9]

Supplementary Table S8. Association of *POU5F1P1* expression and survival in cancer patients (PrognoScan).

Cancer types	N	Cox *p*-value	HR	Endpoint	Dataset	Probe ID
Lung	204	0.005102	0.46	Relapse Free Survival	GSE31210	214532_x_at
	204	0.02751	0.45	Overall Survival	GSE31210	214532_x_at
	41	0.01576	1.72	Overall Survival	GSE11117	H300010272
Ovarian	185	0.017548	0.55	Disease Free Survival	GSE26712	214532_x_at
	185	0.040673	0.61	Overall Survival	GSE26712	214532_x_at
Breast	136	0.011783	1	Distant Metastasis Free Survival	GSE12093	214532_x_at

* "____" represents corrected *p*-value

Supplementary Table S9. Alteration frequency of a three-gene signature (*POU5F1P1*, *PRDM14*, *FAM84B*) in Cancer.

Cancer types	Data source	N	Frequency (%)	Amplification %(N)	Deletion %(N)	Mutation %(N)	Multiple alterations %(N)
NEP	Trento/Comell/ Broad 2016	107	50.47	49.53 (53)	-	0.93 (1)	-
Prostate	FHCRC, 2016	136	48.53	43.38 (59)	2.94 (4)	2.21 (3)	-
Ovarian	TCGA	316	42.41	41.14 (130)	0.63 (2)	0.32 (1)	0.32 (1)
Esophagus	TCGA	184	30.98	27.72 (51)	-	3.26 (6)	0.54 (1)
Breast	METABRIC	2051	28.38	28.38 (582)	-	-	-
The MBC Project	Provisional, October 2017	103	27.18	26.21 (27)	0.97 (1)	-	-
Prostate	SU2C	150	26.67	23.33 (35)	-	2.67 (4)	0.67 (1)
Stomach/ Esophageal	TCGA	265	25.66	23.02 (61)	0.38 (1)	1.51 (4)	0.75 (2)
Breast	TCGA	963	23.26	22.74 (219)	-	0.31 (3)	0.21 (2)
Liver	TCGA	366	21.13	20.66 (44)	-	0.47 (1)	0.27 (1)

Supplementary Table S10. Alteration frequency of a five-gene signature (*POU5F1P1*, *PRDM14*, *FAM84B*, *TCF7L2*, *HLA-C*) in Cancer.

**Cancer types**	**Data source**	**N**	**Frequency (%)**	**Amplification % (N)**	**Deletion % (N)**	**Mutation % (N)**	**Multiple alterations % (N)**
NEP	Trento/Comell/ Broad 2016	107	52.34	50.47 (54)	-	0.93 (1)	0.93 (1)
Prostate	FHCRC, 2016	136	50.74	42.65 (58)	2.94 (4)	4.41 (6)	0.74 (1)
Ovarian	TCGA	316	45.57	43.99 (139)	0.63 (2)	0.63 (2)	0.32 (1)
Esophagus	TCGA	184	33.70	28.26 (52)	-	4.89 (9)	0.54 (1)
Stomach/ Esophageal	TCGA	265	33.21	27.55 (73)	0.75 (2)	3.4 (9)	1.51 (4)
The MBC Project	Provisional, October 2017	103	32.04	31.07 (32)	0.97 (1)	-	-
Breast	METABRIC	2051	29.11	29.11 (597)	-	-	-
Prostate	SU2C	150	28.67	24.67 (37)	0.67 (1)	2.67 (4)	0.67 (1)
Breast	TCGA	963	24.92	23.05 (222)	0.42 (4)	1.04 (10)	0.42 (4)
Liver	TCGA	366	23.52	19.67 (72)	0.82 (3)	2.73 (10)	0.27 (1)
Pancreas	UTSW	109	22.94	19.27 (21)	1.83 (2)	1.83 (2)	-
Stomach	TCGA	393	22.39	13.99 (55)	0.76 (3)	6.36 (25)	1.27 (5)
Colorectal	TCGA	220	20.45	8.64 (19)	2.73 (6)	9.09 (20)	-

Supplementary Table S11. Percentages of alterations in *POU5F1P1*, *PRDM14*, and *FAM84B*.

**Cancer types**	***POU5F1P1***	***PRDM14***	***FAM84B***
NEP	52%	53%	53%
Prostate	44%	28%	33%
Ovarian	40%	9%	39%
Esophagus	28%	6%	24%
Breast	26%	16%	25%
The MBC Project	26%	8%	26%
Prostate	13%	18%	13%
Stomach/Esophageal	23%	6%	17%
Breast	22%	10%	20%
Liver	18%	12%	18%

Supplementary Table S12. Percentages of alterations in *POU5F1P1*, *PRDM14*, *FAM84B*, *TCF7L2*, and *HLA-C*.

**Cancer types**	***POU5F1P1***	***PRDM14***	***FAM84B***	***TCF7L2***	***HLA-C***
NEP	52.0%	53.0%	53.0%	8.0%	21%
Prostate	44.0%	28.0%	33.0%	4.0%	6%
Ovarian	40.0%	9.0%	39.0%	3.0%	0%
Esophagus	28.0%	6.0%	24.0%	1.1%	1.6%
Stomach/Esophageal	23.0%	6.0%	17.0%	4.0%	7%
The MBC Project	26.0%	8.0%	26.0%	9.0%	1.3%
Breast	26.0%	16.0%	25.0%	0.3%	1%
Prostate	13.0%	18.0%	13.0%	2.7%	0%
Breast	22.0%	10.0%	20.0%	1.2%	0.6%
Liver	18.0%	12.0%	18.0%	1.9%	0.3%
Pancreas	14.0%	5.0%	14.0%	1.8%	10%
Stomach	14.0%	5.0%	10.0%	5.0%	1.8%
Colorectal	7.0%	6.0%	6.0%	10.0%	0.9%

Supplementary Table S13. *POU5F1P3* expression in each Cancer types type.

Cancer types	Cancer subtype	*p*-value	Fold change	Rank (%)	Sample	Reference
Breast	Invasive Breast Carcinoma Stroma	3.49E-22	-3.871	5	59	[3]
Colorectal	Rectal Adenocarcinoma	8.66E-23	2.608	4	130	[5]
	Rectal Adenocarcinoma	1.06E-10	2.441	11	237	[17]
	Colon Adenocarcinoma	5.04E-11	2.485	11	237	[17]
	Colorectal Carcinoma	9.67E-05	1.143	14	105	[18]
	Colorectal Carcinoma	2.17E-04	3.414	23	82	[19]
	Cecum Adenocarcinoma	1.58E-04	1.908	22	237	[17]
Kidney	Hereditary Clear Cell Renal Cell Carcinoma	2.02E-12	2.508	2	70	[15]
	Non-Hereditary Clear Cell Renal Cell Carcinoma	1.88E-06	2.021	8	70	[15]
Other	Seminoma, NOS	2.03E-09	20.006	2	107	[9]
	Mixed Germ Cell Tumor, NOS	2.24E-09	4.98	6	107	[9]
	Embryonal Carcinoma, NOS	1.78E-06	9.725	9	107	[9]

Supplementary Table S14. Association of *POU5F1P3* expression and survival in cancer patients (PrognoScan).

**Cancer types**	**N**	**Cox *p*-value**	**HR**	**Endpoint**	**Dataset**	**Probe ID**
Blood	559	0.002145	0.71	Disease Specific Survival	GSE2658	210265_x_at
Breast	77	0.016679	86.7	Relapse Free Survival	GSE9195	210265_x_at
Lung	117	0.023852	0.67	Overall Survival	GSE13213	A_24_P383640
	138	0.049494	0	Relapse Free Survival	GSE8894	210265_x_at
Eye	63	<u>0.024368</u>	3.23	Distant Metastasis Free Survival	GSE22138	210265_x_at
Head & Neck	28	<u>0.037561</u>	0.52	Relapse Free Survival	GSE2837	g12382251_3p_x_at

Supplementary Table S15. *POU5F1P4* expression in each Cancer types.

**Cancer types**	**Cancer subtype**	***p*-value**	**Fold change**	**Rank (%)**	**Sample**	**Reference**
Brain and CNS	Oligodendroglioma	6.33E-06	2.537	9	180	[20]
	Glioblastoma	1.95E-04	1.932	23	180	[20]
Breast	Invasive Breast Carcinoma Stroma	9.42E-22	-13.876	6	59	[3]
Colorectal	Rectal Adenocarcinoma	1.44E-20	2.188	5	130	[5]
	Colon Adenocarcinoma	8.83E-05	1.489	14	105	[6]
	Rectosigmoid Adenocarcinoma	1.23E-04	1.443	8	105	[6]
	Colon Adenocarcinoma	2.98E-09	1.918	15	237	[17]
	Colorectal Carcinoma	5.75E-05	3.055	20	82	[19]
	Rectal Adenocarcinoma	1.31E-07	1.751	17	237	[17]
Gastric	Diffuse Gastric Adenocarcinoma	8.17E-07	-2.582	1	69	[21]
Kidney	Clear Cell Renal Cell Carcinoma	1.85E-05	4.847	6	20	[7]
	Hereditary Clear Cell Renal Cell Carcinoma	6.51E-08	2.374	7	70	[15]
	Non-Hereditary Clear Cell Renal Cell Carcinoma	3.31E-05	1.835	11	70	[15]
	Papillary Renal Cell Carcinoma	3.29E-05	1.359	16	92	[8]
Other	Seminoma, NOS	2.25E-10	14.173	1	107	[9]
	Mixed Germ Cell Tumor, NOS	1.06E-09	4.573	5	107	[9]
	Embryonal Carcinoma, NOS	1.80E-06	7.915	9	107	[9]

Supplementary Table S16. Association of *POU5F1P4* expression and survival in cancer patients (PrognoScan).

Cancer types	N	Cox *p*-value	HR	Endpoint	Dataset	Probe ID
Breast	77	0.046795	28.12	Relapse Free Survival	GSE9195	210905_x_at
	115	0.005326	5.38	Distant Metastasis Free Survival	GSE19615	210905_x_at
	125	0.049525	0.76	Distant Metastasis Free Survival	GSE2990	210905_x_at
Ovarian	185	0.027097	0.52	Overall Survival	GSE26712	210905_x_at
	185	0.00484	0.47	Disease Free Survival	GSE26712	210905_x_at
Head & Neck	28	0.022458	1.41	Relapse Free Survival	GSE2837	g12382245_3p_x_at
Lung	129	0.013688	0.98	Overall Survival	GSE4573	210905_x_at
	82	0.00535	5.95	Overall Survival	jacob-00182-CANDF	210905_x_at

Supplementary Figures

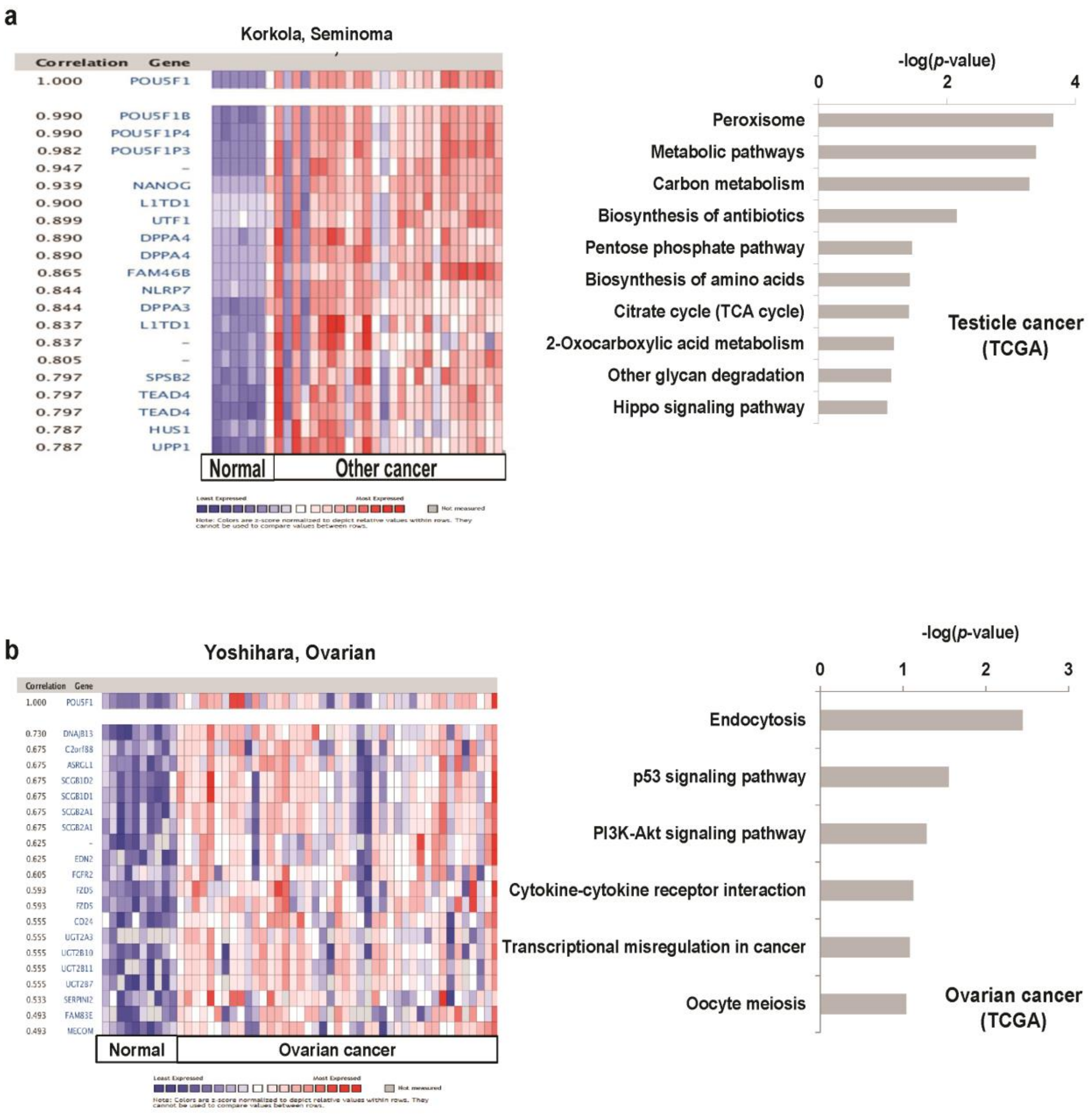

Supplementary Figure S1. Co-expression pattern of *POU5F1* in several cancer types: *OCT4* (*POU5F1*) was co-expressed with the indicated genes across a panel of across 26 other cancer and 7 normal samples (**a,** left panel) and 43 ovarian cancer and 10 normal samples (**b**, left panel). Bar length represents the significance and negative logarithm of enrichment *p*-value. The co-expressed genes were retrieved from TCGA database using cBioPortal web. Using the co-expressed genes list, top 10 significant GO processes were confirmed using DAVID functional annotation in testicle (**a,** right panel) and ovarian (**b**, right panel) cancer. Bar length represents the significance and negative logarithm of enrichment *p*-value.

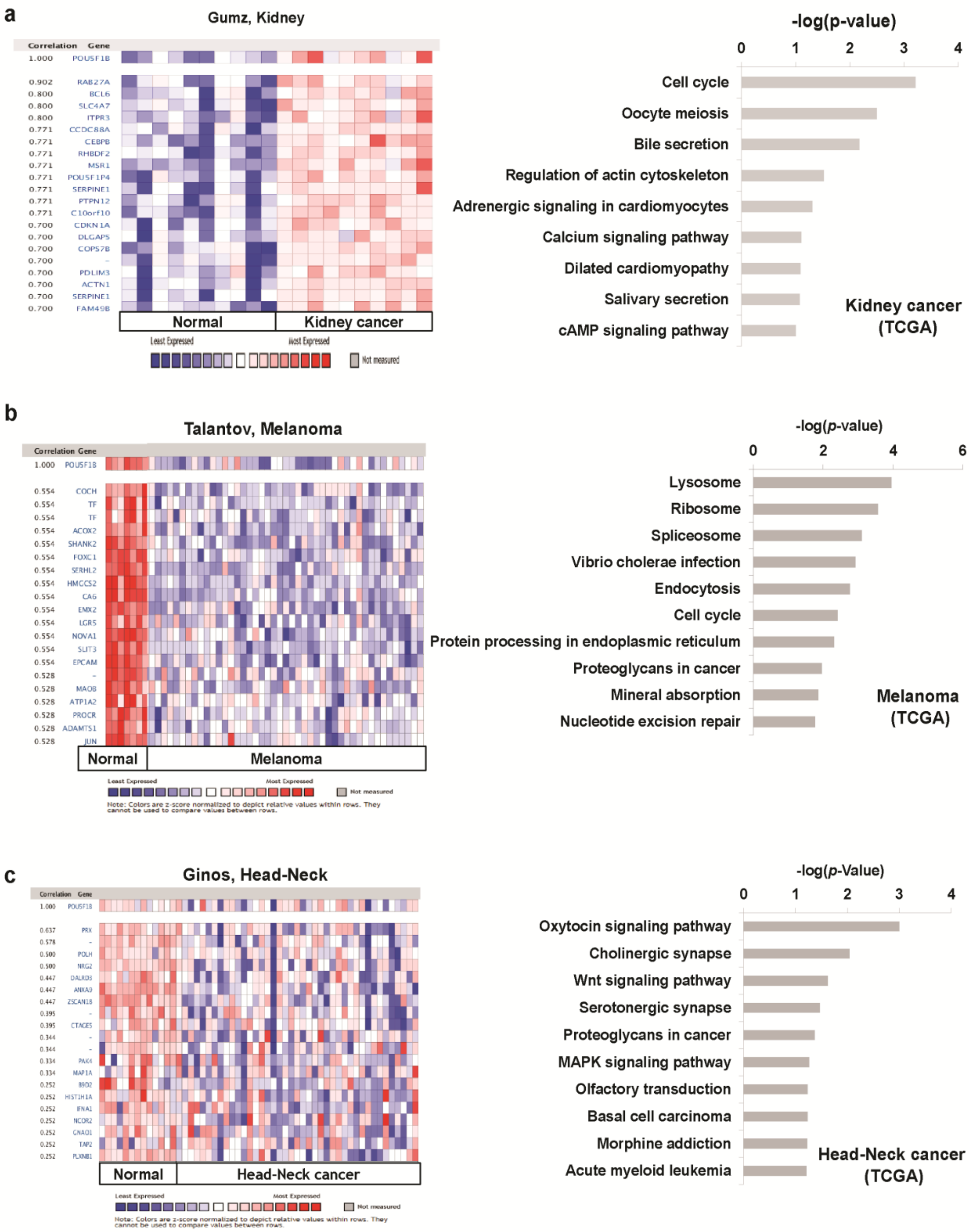
a
Gumz, Kidney
Normal
Kidney cancer
-log(p-value)
Cell cycle
Oocyte meiosis
Bile secretion
Regulation of actin cytoskeleton
Adrenergic signaling in cardiomyocytes
Calcium signaling pathway
Dilated cardiomyopathy
Salivary secretion
cAMP signaling pathway
Kidney cancer (TCGA)
b
Talantov, Melanoma
Normal
Melanoma
-log(p-value)
Lysosome
Ribosome
Spliceosome
Vibrio cholerae infection
Endocytosis
Cell cycle
Protein processing in endoplasmic reticulum
Proteoglycans in cancer
Mineral absorption
Nucleotide excision repair
Melanoma (TCGA)
c
Ginos, Head-Neck
Normal
Head-Neck cancer
-log(p-Value)
Oxytocin signaling pathway
Cholinergic synapse
Wnt signaling pathway
Serotonergic synapse
Proteoglycans in cancer
MAPK signaling pathway
Olfactory transduction
Basal cell carcinoma
Morphine addiction
Acute myeloid leukemia
Head-Neck cancer (TCGA)

Supplementary Figure S2. Co-expression pattern of *POU5F1P1* in various cancer types: *POU5F1P1* was co-expressed with the presented genes across a panel of 42 kidney cancer and 11 normal samples (**a,** left panel). In a panel of 45 melanoma and 7 normal samples, *POU5F1P1* was co-expressed with the presented genes (**b,** left panel). *POU5F1P1* was also co-expressed with the presented genes across 42 head-neck cancer and 13 normal sample (**c,** left panel). Bar length represents the significance and negative logarithm of enrichment *p*-value. The co-expressed genes were retrieved from TCGA database using cBioPortal web. Using the co-expressed genes list, top 10 significant GO processes were confirmed using DAVID functional annotation in kidney (**a,** right panel), melanoma (**b**, right panel) and head-neck (**c**, right panel) cancer. Bar length represents the significance and negative logarithm of enrichment *p*-value.

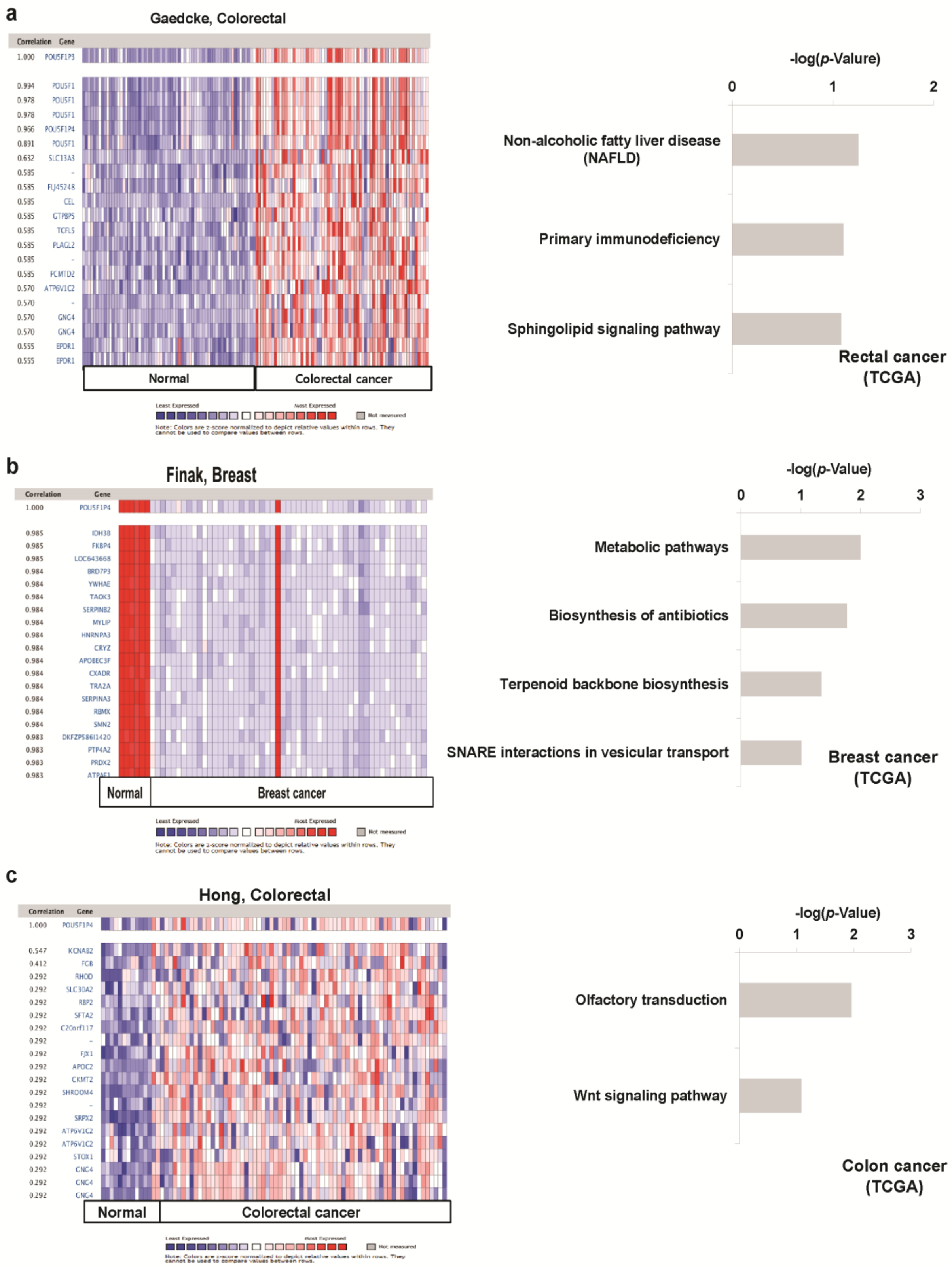
a
Gaedcke, Colorectal
Correlation Gene
Normal
Colorectal cancer
Least Expressed
Most Expressed
Not measured
-log(p-Valure)
0
1
2
Non-alcoholic fatty liver disease (NAFLD)
Primary immunodeficiency
Sphingolipid signaling pathway
Rectal cancer (TCGA)
b
Finak, Breast
Correlation Gene
Normal
Breast cancer
Least Expressed
Most Expressed
Not measured
-log(p-Value)
0
1
2
3
Metabolic pathways
Biosynthesis of antibiotics
Terpenoid backbone biosynthesis
SNARE interactions in vesicular transport
Breast cancer (TCGA)
c
Hong, Colorectal
Correlation Gene
Normal
Colorectal cancer
Least Expressed
Most Expressed
Not measured
-log(p-Value)
0
1
2
3
Olfactory transduction
Wnt signaling pathway
Colon cancer (TCGA)

Supplementary Figure S3. Co-expression pattern of POU5F1P3 and POU5F1P4 in various cancer types: *POU5F1P3* was co-expressed with the presented genes across a panel of 65 colorectal cancer and 65 normal samples (**a,** left panel). *POU5F1P4* was co-expressed with the indicated genes across a panel of 53 breast cancer and 6 normal sample and 70 colorectal cancer and 12 normal samples (**b** and **c,** left panel). Bar length represents the significance and negative logarithm of enrichment *p*-value. The co-expressed genes were retrieved from TCGA database using cBioPortal web. Using the co-expressed genes list, top significant GO processes related to POU5F1P3 were confirmed using DAVID functional annotation in colorectal cancer (a, right panel). Significant GO processes related to POU5F1P4 were also confirmed using DAVID functional annotation in breast (**b**, right panel) and colorectal (**c**, right panel) cancer. Bar length represents the significance and negative logarithm of enrichment *p*-value.

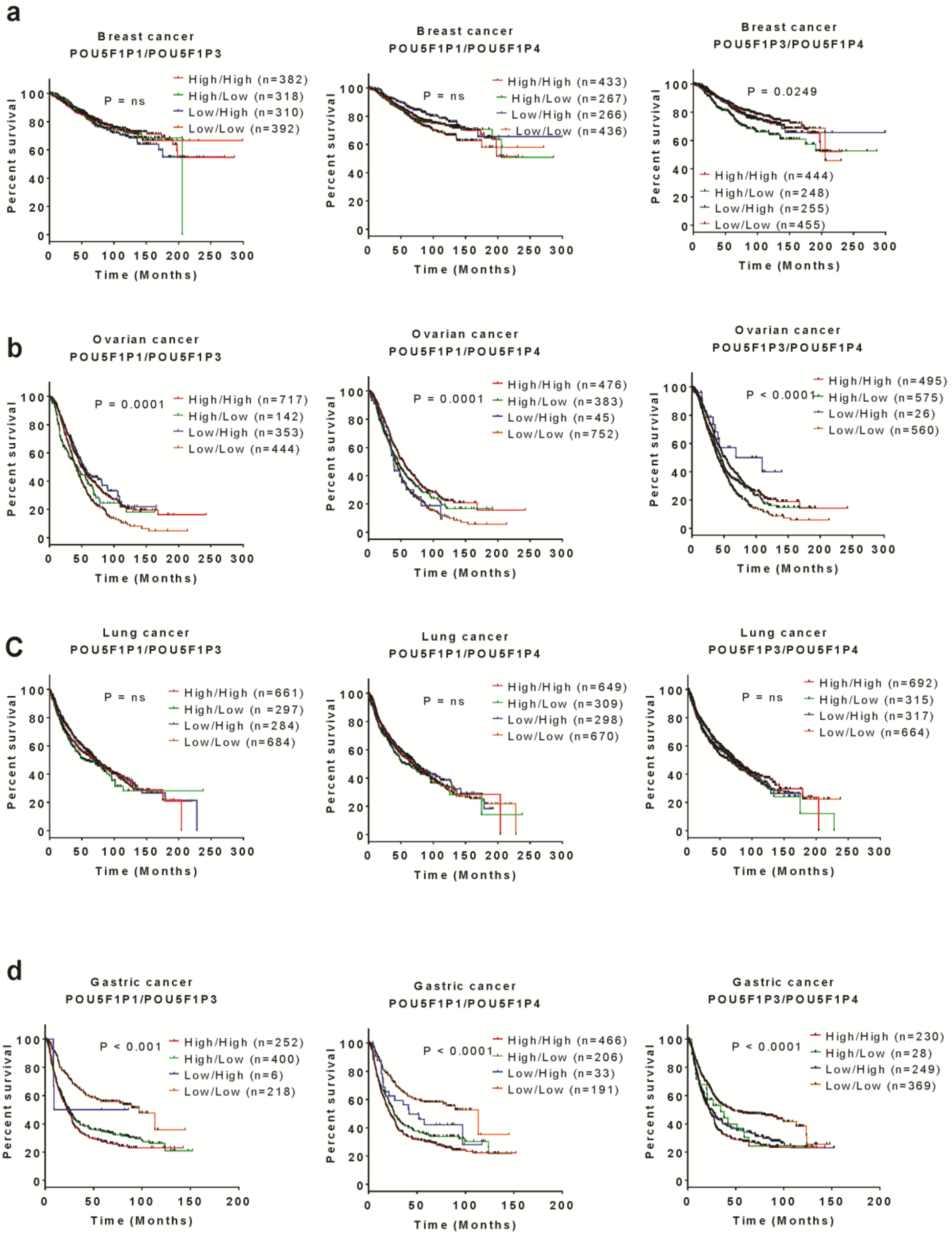
a
Breast cancer
POU5F1P1/POU5F1P3
P = ns
High/High (n=382)
High/Low (n=318)
Low/High (n=310)
Low/Low (n=392)
Breast cancer
POU5F1P1/POU5F1P4
P = ns
High/High (n=433)
High/Low (n=267)
Low/High (n=266)
Low/Low (n=436)
Breast cancer
POU5F1P3/POU5F1P4
P = 0.0249
High/High (n=444)
High/Low (n=248)
Low/High (n=255)
Low/Low (n=455)
b
Ovarian cancer
POU5F1P1/POU5F1P3
P = 0.0001
High/High (n=717)
High/Low (n=142)
Low/High (n=353)
Low/Low (n=444)
Ovarian cancer
POU5F1P1/POU5F1P4
P = 0.0001
High/High (n=476)
High/Low (n=383)
Low/High (n=45)
Low/Low (n=752)
Ovarian cancer
POU5F1P3/POU5F1P4
P < 0.0001
High/High (n=495)
High/Low (n=575)
Low/High (n=26)
Low/Low (n=560)
C
Lung cancer
POU5F1P1/POU5F1P3
P = ns
High/High (n=661)
High/Low (n =297)
Low/High (n=284)
Low/Low (n=684)
Lung cancer
POU5F1P1/POU5F1P4
P = ns
High/High (n=649)
High/Low (n=309)
Low/High (n=298)
Low/Low (n=670)
Lung cancer
POU5F1P3/POU5F1P4
P = ns
High/High (n=692)
High/Low (n=315)
Low/High (n=317)
Low/Low (n=664)
d
Gastric cancer
POU5F1P1/POU5F1P3
P < 0.001
High/High (n=252)
High/Low (n=400)
Low/High (n=6)
Low/Low (n=218)
Gastric cancer
POU5F1P1/POU5F1P4
P < 0.0001
High/High (n=466)
High/Low (n=206)
Low/High (n=33)
Low/Low (n=191)
Gastric cancer
POU5F1P3/POU5F1P4
P < 0.0001
High/High (n=230)
High/Low (n=28)
Low/High (n=249)
Low/Low (n=369)
Percent survival
Time (Months)

Supplementary Figure S4. Expression co-occurrence of *OCT4* pseudogenes in relation to the clinical prognosis of cancer patients. The multivariate survival curves compare the clinical prognosis in patients with high/high (red), high/low (green), low/high (blue), and low/low (orange) expression co-occurrence of *POU5F1P1*/*POU5F1P3*, *POU5F1P1*/*POU5F1P4*, and *POU5F1P3*/*POU5F1P4* in breast (**a**), ovarian (**b**), lung (**c**) and gastric (**d**) cancers. The clinical outcome data were retrieved from the Kaplan-Meier plotter database. Information indicating statistical significance represents p < 0.05, and a non-significant p-value is expressed as 'ns' in the graph.

Supplementary References

1. Modlich, O. *et al.* Identifying superficial, muscle-invasive, and metastasizing transitional cell carcinoma of the bladder: use of cDNA array analysis of gene expression profiles. *Clin. Cancer types Res.* **10**, 3410-3421 (2004).
2. Sanchez-Carbayo, M., Socci, N. D., Lozano, J., Saint, F. & Cordon-Cardo, C. Defining molecular profiles of poor outcome in patients with invasive bladder Cancer types using oligonucleotide microarrays. *J. Clin. Oncol.* **24**, 778-789 (2006).
3. Finak, G. *et al.* Stromal gene expression predicts clinical outcome in breast Cancer types. *Nat. Med.* **14**, 518 (2008).
4. Ma, X.-J., Dahiya, S., Richardson, E., Erlander, M. & Sgroi, D. C. Gene expression profiling of the tumor microenvironment during breast Cancer types progression. *Breast Cancer types Res.* **11**, R7 (2009)..
5. Gaedcke, J. *et al.* Mutated KRAS results in overexpression of DUSP4, a MAP-kinase phosphatase, and SMYD3, a histone methyltransferase, in rectal carcinomas. *Genes Chromosomes Cancer types* **49**, 1024-1034 (2010)..
6. Kaiser, S. *et al.* Transcriptional recapitulation and subversion of embryonic colon development by mouse colon tumor models and human colon Cancer types. *Genome Biol. Evol.* **8**, R131 (2007).
7. Gumz, M. L. *et al.* Secreted frizzled-related protein 1 loss contributes to tumor phenotype of clear cell renal cell carcinoma. *Clin. Cancer types Res.* **13**, 4740-4749 (2007).
8. Jones, J. *et al.* Gene signatures of progression and metastasis in renal cell Cancer types. *Clin. Cancer types Res.* **11**, 5730-5739 (2005).
9. Korkola, J. E. *et al.* Down-regulation of stem cell genes, including those in a 200-kb gene cluster at 12p13. 31, is associated with in vivo differentiation of human male germ cell tumors. *Cancer types Res.* **66**, 820-827 (2006).
10. Skotheim, R. I. *et al.* Differentiation of human embryonal carcinomas in vitro and in vivo reveals expression profiles relevant to normal development. *Cancer types Res.* **65**, 5588-5598 (2005).
11. Yoshihara, K. *et al.* Gene expression profiling of advanced-stage serous ovarian Cancer typess distinguishes novel subclasses and implicates ZEB2 in tumor progression and prognosis. *Cancer types Sci.* **100**, 1421-1428 (2009).
12. Hendrix, N. D. *et al.* Fibroblast growth factor 9 has oncogenic activity and is a downstream target of Wnt signaling in ovarian endometrioid adenocarcinomas. *Cancer types Res.* **66**, 1354-1362 (2006).
13. Detwiller, K. Y. *et al.* Analysis of hypoxia-related gene expression in sarcomas and effect of hypoxia on RNA interference of vascular endothelial cell growth factor A. *Cancer types Res.* **65**, 5881-5889 (2005).
14. Barretina, J. *et al.* Subtype-specific genomic alterations define new targets for soft-tissue sarcoma therapy. *Nat. Genet.* **42**, 715 (2010).
15. Beroukhim, R. *et al.* Patterns of gene expression and copy-number alterations in von-hippel lindau

disease-associated and sporadic clear cell carcinoma of the kidney. *Cancer types Res.* **69**, 4674-4681 (2009).

16. Talantov, D. *et al.* Novel genes associated with malignant melanoma but not benign melanocytic lesions. *Clin. Cancer types Res.* **11**, 7234-7242 (2005).
17. Weinstein, J. N. *et al.* The Cancer types genome atlas pan-Cancer types analysis project. *Nat. Genet.* **45**, 1113 (2013).
18. Skrzypczak, M. *et al.* Modeling oncogenic signaling in colon tumors by multidirectional analyses of microarray data directed for maximization of analytical reliability. *Plos One* **5**, e13091 (2010)..
19. Hong, Y., Downey, T., Eu, K. W., Koh, P. K. & Cheah, P. Y. A ‘metastasis-prone’signature for early-stage mismatch-repair proficient sporadic colorectal Cancer types patients and its implications for possible therapeutics. *Clin. Exp. Metastasis* **27**, 83-90 (2010).
20. Sun, L. *et al.* Neuronal and glioma-derived stem cell factor induces angiogenesis within the brain. *Cancer types cell* **9**, 287-300 (2006).
21. D’Errico, M. *et al.* Genome-wide expression profile of sporadic gastric Cancer typess with microsatellite instability. *Eur. J. Cancer types* **45**, 461-469 (2009).
